# Three-Tier Prognostic Stratification of Lung Carcinoids (NET G1-G2-G3) by Multivariable, Data-Driven Integration of Ki-67 and Mitotic Count

**DOI:** 10.1007/s12022-026-09920-4

**Published:** 2026-05-21

**Authors:** Giulia Orlando, Valentina Veronesi, Eleonora Duregon, Vanessa Zambelli, Francesco Leo, Elisa Carla Fontana, Enrico Ruffini, Luisella Righi, Giuseppe Pelosi, Marco Volante, Mauro Papotti

**Affiliations:** 1 Division of Pathology, City of Health and Sciences University Hospital, Department of Oncology, University of Turin, Turin, Italy; 2 Department of Biomedical, Surgical and Dental Sciences, University of Milan, Milan, Italy; 3 Division of Pathology, Department of Oncology, San Luigi University Hospital, University of Turin, Orbassano, Italy; 4 Division of Thoracic Surgery, San Luigi University Hospital, University of Turin, Orbassano, Italy; 5 Division of Thoracic Surgery, City of Health and Sciences University Hospital, University of Turin, Turin, Italy; 6 Department of Oncology and Hemato-Oncology, University of Milan, Milan, Italy; 7 Division of Pathology, San Luigi University hospital, Regione Gonzole 10, Orbassano, Torino, 10043 Italy; 8 Inter-Hospital Division of Pathology, IRCCS MultiMedica, Via Gaudenzio Fantoli, 16/15, Milan, 20138 Italy

**Keywords:** Lung, Carcinoid, Neuroendocrine tumor, Classification, Ki-67 index, Disease-free survival

## Abstract

Pulmonary neuroendocrine tumors (NETs) include typical and atypical carcinoids, corresponding to low- and intermediate-grade malignancies. Integration of necrosis, mitotic count per 2 mm² (MC), and Ki-67 index, a desirable criterion in the current WHO scheme, may enable a classification framework beyond conventional histology. We used a clustering method for mixed-type data, including necrosis, MC and Ki-67 index, on a single-institution cohort of 358 typical and 125 atypical carcinoids with long-term follow-up. An external validation series of 259 additional carcinoids was used. A three-cluster solution was selected, yielding a tiered prognostic stratification model, that included NETG1 (383 cases; mean MC: 0.7; necrosis: 8.1% of cases; mean Ki-67 index: 2.5%), NETG2 (77 cases; mean MC 2.3; necrosis 27.3%; mean Ki-67: 11.4%), and NETG3 (23 cases; mean MC: 5.5; necrosis 60.9%; mean Ki-67: 25.1%). The model was independent of histological classification. Ki-67 index was the strongest discriminator, distinguishing NET G1 tumors with a Ki-67 index < 6% from NET G3 tumors (Ki-67 > 16%), while MC contributed to identifying a subset of NET G2 tumors. The selected cut-offs were derived through a statistical approach evaluating hundreds of decision-tree configurations, following a sequential algorithm from Ki-67 to MC, with 6–16% identified as the most informative Ki-67 range for intermediate stratification. Necrosis added no discriminative value, as it was closely correlated with the Ki-67 index and MC. This classification corresponded to a progressive worsening of overall survival and, more significantly, relapse-free survival, and it was transferable to the external cohort. In conclusion, based on routinely available pathological parameters, this model provides a practical and reproducible classification of resected pulmonary NETs, overcoming the traditional typical–atypical carcinoid dichotomy and is potentially applicable to biopsy material.

**Supplementary Information:**

The online version contains supplementary material available at 10.1007/s12022-026-09920-4.

## Introduction

According to the current WHO classification of pulmonary neuroendocrine neoplasms (NENs) [1], well-differentiated neuroendocrine tumors (NETs) are distinguished from poorly differentiated neuroendocrine carcinomas (NECs). The former includes typical and atypical carcinoids (TC and AC), corresponding to Grade 1 and Grade 2 tumors, respectively. The latter comprise large cell neuroendocrine carcinoma (LCNEC) and small cell lung carcinoma (SCLC), which are by definition high-grade (Grade 3) lesions. Combined carcinomas (with adeno- or squamous components) are recognized as a separate category (Table 1). In small samples of primary and metastatic tumors, subtyping of carcinoid/NET should be avoided, and the diagnosis should be reported as “carcinoid/NET, not otherwise specified (NOS)” and “metastatic carcinoid NOS” [1].

**Table 1:** The spectrum of lung neuroendocrine neoplasms: clinico-pathological details

	Typical carcinoid-TC/NET G1^†^	Atypical carcinoid-AC/NET G2^†^	Carcinoid tumor with elevated proliferation rates and/or mitoses*	LCNEC	SCLC
Diagnostic criteria					
Mitoses per 2 mm^2^	0–1	2–10	> 10	> 10 (median: 70)	> 10 (median: 80)
Necrosis	No	Focal, if any	Focal	Extensive	Extensive to geographic
Neuroendocrine differentiation	Well-differentiated	Well-differentiated	Well-differentiated	Poorly differentiated	Poorly differentiated
Neuroendocrine markers	+++	++/+++	++/+++	++	+/±/-
Ki-67 labeling index	Up to 5%	Up to 30%	> 30%**	40–80%	80–100%
Tumor grade	Low grade	Intermediate grade	Intermediate between AC and NEC	High grade	High grade
Tumor prognosis	Good	Intermediate	Worse than AC	Poor	Poor
Combined with NSCC component	Very rare	Very rare	Not reported, but possible	Up to 25% of resected LCNEC	Up to 25% of resected SCLC
Cytology or biopsy samples					
Primary	Carcinoid tumor/NET NOS	Carcinoid tumor/NET NOS	Carcinoid with elevated proliferation	NSCC with features of LCNEC	SCLC
Metastasis	Metastatic carcinoid NOS	Metastatic carcinoid NOS	Carcinoid with elevated proliferation	NSCC with features of LCNEC	SCLC

NET: neuroendocrine tumor, *LCNEC* large cell neuroendocrine carcinoma, *SCLC* small cell carcinoma of the lung, *NSCC* non-small cell carcinoma, *NOS* not otherwise specified. ^†^: Diagnostic criteria for typical and atypical carcinoid are applicable to lung resection specimens only. *: provisional category included in the 2021 WHO classification. Neuroendocrine markers usually include synaptophysin, chromogranin A and INSM1. **: the WHO classification mandates a Ki-67 cut-off > 30% for identifying proliferating carcinoids

The current diagnostic criteria of carcinoids/NETs are less straightforward and reproducible, and this group of NENs is far from being homogenous, posing challenges for therapeutic decision-making. According to the histological diagnostic criteria established by the 2021 WHO classification [2], TC and AC are defined by two parameters, i.e., mitotic count (MC) and presence of necrosis, while other features, including growth pattern, cell size, atypia, histologic variants, nodal metastases, may impact the differential diagnosis, but have limited clinical significance.

An accurate prediction of clinical outcome in NETs remains difficult to achieve, despite different attempts based on refined morphology and staging, multi-marker profiling, and molecular classifications [3–20]. The Ki-67 proliferation index (hereafter, simply Ki-67) is probably the most extensively investigated prognostic factor in lung NENs [21–26]. Different cut-offs have been investigated, and a threshold around 5% (rather than 3%) has been suggested in several studies [21, 22, 26–31] to better stratify tumor malignancy and predict relapse-free survival (RFS), with higher values generally associating to an AC diagnosis. Nevertheless, Ki-67 was not incorporated as an essential criterion into the 2021 WHO classification, as its reproducibility on resected specimens is still deemed controversial [5]. This contrasts with its essential role in the gastroenteropancreatic (GEP) NETs that are embryologically related neoplasms (at least with regard to gastric and pancreatic NENs) [32]. In the GEP NETs, MC and Ki-67 are the two parameters used to stratify three tumor grades, while necrosis has not been considered, although it may bear prognostic relevance [33]. Furthermore, Ki-67 is prognostic also in other NENs, including parathyroid tumors [34, 35], and medullary thyroid carcinoma, where a 5% cut-off helps distinguish low from high-grade forms [36, 37].

This study aimed to investigate a large, mono-institutional series of resected lung carcinoids/NETs to determine whether integrating Ki-67 into the current use of MC and necrosis could improve stratification into clinically meaningful prognostic groups. Using a multivariable, data-driven analytical approach, we show that Ki-67 assessment expands traditional classification parameters (MC and necrosis) and identifies relatively homogeneous subgroups of carcinoids/NETs beyond the current TC/AC dichotomy.

## Materials and Methods

### Case Series

From the pathology files of the City of Health & Sciences and San Luigi di Orbassano University Hospitals in Turin, Italy, a consecutive series of 525 resected primary lung carcinoids diagnosed between 1990 and 2024 was retrospectively selected, after obtaining the ethical approval (project GranCapo, #0003323 of July 22, 2025). Among these, 483 cases were included in the present study, based on the availability of survival data and sufficient residual tissue material, comprising 358 TC and 125 AC. Three AC cases fulfilled criteria for “carcinoid/NET with elevated MC and/or Ki-67 proliferation index” [2], two of them had Ki-67 = 40 and 45%, while the third had MC = 16. Clinico-pathological parameters included gender, age at diagnosis, tumor size, location, stage, pT and pN (according to both 8th and 9th American Joint Committee on Cancer editions), mitosis/2 mm^2^, necrosis, Ki-67, tumorlets, multifocality, spread through air spaces (STAS), vascular invasion and follow-up status, which were collected and recorded in an anonymized database. MC was assessed on hematoxylin and eosin-stained (H&E) slides in areas of highest mitotic activity. Counts were expressed as the number of mitoses per 2 mm², in accordance with WHO recommendations, ensuring standardized assessment across cases. The Ki-67 labeling index (hereinafter referred to as Ki-67) was assessed according to European Neuroendocrine Tumor Society (ENETS) recommendations for gastrointestinal neuroendocrine tumors [38]. The index was calculated as the percentage of positively stained nuclei among at least 2000 tumor cells, counted in areas showing the highest labeling (“hot spots”). Prior to data collection, all cases were de-identified and coded by a pathology staff member not involved in the study, and all data were accessed anonymously. An external series of further 253 carcinoids with publicly available data [9] was used for validation.

### Statistical Analysis

The relationship between necrosis and proliferation markers was compared using Wilcoxon-Mann-Whitney (WMW) tests and Hodges-Lehmann estimates of location shift. The KAMILA algorithm for mixed-type data (numerical and categorical) was used to jointly cluster necrosis, MC, and Ki-67, thereby deriving an alternative, data-driven grouping of NETs beyond the WHO two-tier (TC vs. AC) scheme [38, 39]. To translate the data-driven clusters into clinically usable thresholds, we fitted conditional inference trees [40] to predict cluster labels from Ki-67, MC, and necrosis. Overall survival (OS) and RFS analyses were restricted to patients with complete status information and last follow-up/death dates and analyzed using Cox models and Kaplan-Meier (KM) curves. All statistical analyses were performed using R software [41], version 4.1.2. P-values were 2-sided, and the base significance level was defined as α < 0.05. Further details on the statistical methods used in the present study are presented in the Supplementary Methods, Section b) Statistical analysis.

## Results

### Sample Description and Tumor Parameters Distributions

Clinico-pathological features of the 483 resected primary lung carcinoids are summarized in Supplementary Table 1. During follow-up, relapse occurred in 58 patients (12%). At the last follow-up, 70.8% of patients were alive with no evidence of disease (NED), whereas 8.9% had died of disease (DOD). Necrosis was uncommon (absent in 86.3% of cases) and, when present, occurred in tumors with higher proliferative activity, correlating with Ki-67 rather than MC (Supplementary Table 2; Supplementary Fig. 1; Supplementary Results 1, Sections a and b). A non-trivial subset of tumors (*n* = 41) lacked necrosis and had < 2 mitoses per 2 mm^2^, thus were still classified as TC under current WHO criteria, but showed mid-range Ki-67 values (i.e., in the visually identified range: 6–15%). This pattern suggests that relying primarily on necrosis may overlook clinically relevant variation in Ki-67, especially among necrosis-negative tumors, supporting the rationale for incorporating it when defining data-driven groups.

## Alternative Grouping for NETs

**Fig. 1:**
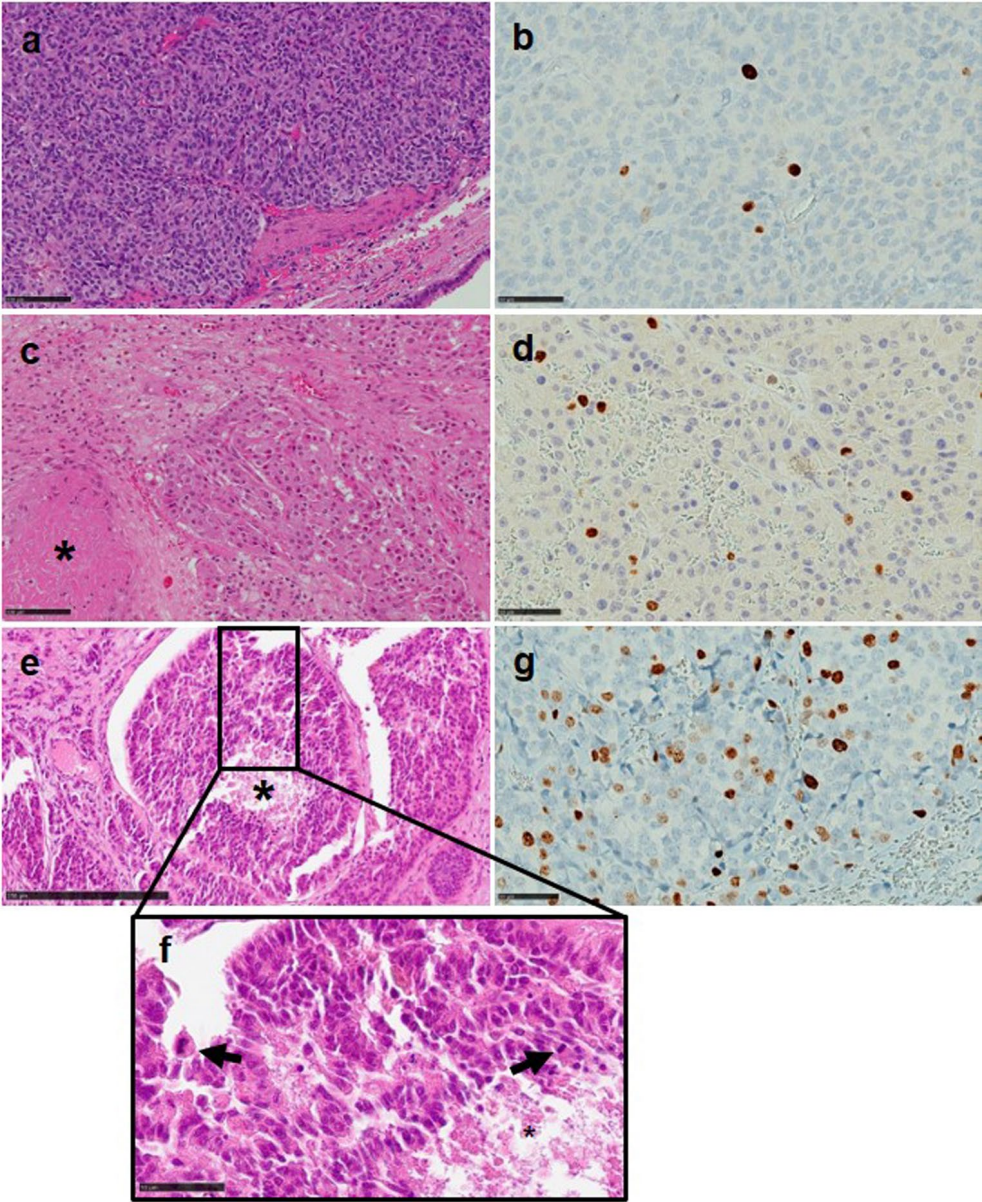
Representative images of lung Carcinoids/NET G1, G2 and G3 (**a**, **c**, **e**-**f**), with necrotic foci (* in **c**, **e**, **f**), mitotic figures (arrows in **f**) and a Ki-67 index of 2%, 7% and 28% (**b**, **d**, **g**), respectively

**Fig. 2:**
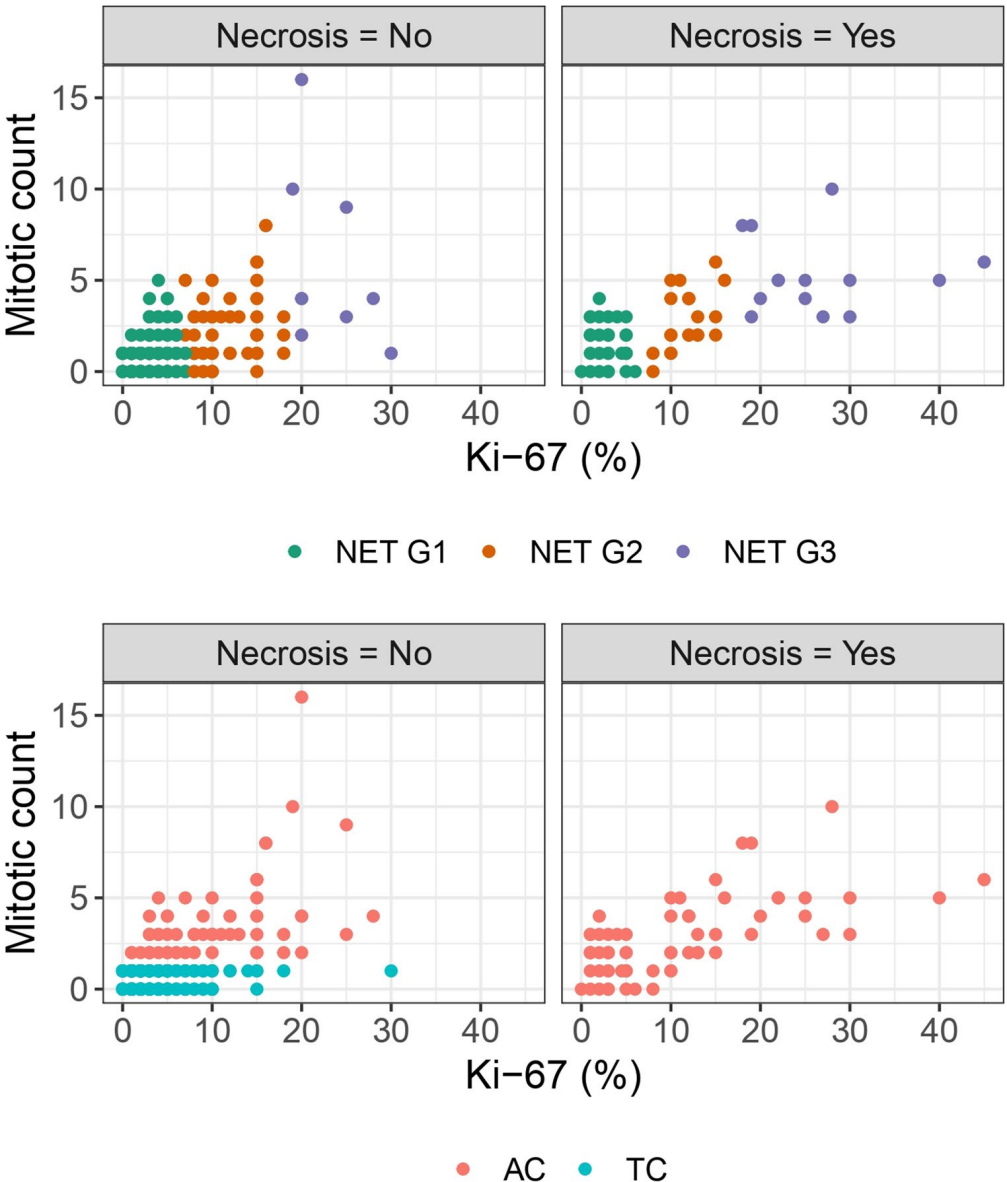
Scatterplots of Ki-67 (%) versus mitotic count, separated by necrosis status. Top: Points are colored by the 3-cluster solution and labeled as NET G3, NET G2, and NET G1, showing how the unsupervised groups distribute across Ki-67 and mitotic count within each necrosis stratum. Bottom: The same panels, but points are colored by the current WHO classification (AC vs. TC)

Table 2 shows the KAMILA cluster centers for K = 3, and their correspondence with the current WHO classification. Clusters were sequentially named Carcinoid/NET G1, Carcinoid/NET G2, and Carcinoid/NET G3, to reflect progressive aggressiveness **(**Fig. 1**)**. Carcinoid/NET G1 group (*N* = 383) contained the least proliferative tumors (mean Ki-67: 2.5% [SD 1.7]; mean MC: 0.7 [SD 0.8]), largely necrosis-negative (91.9%), although a small number of them – classified as ACs by WHO criteria– harbored necrosis. Carcinoid/NET G2 group (*N* = 77) showed intermediate values (mean Ki-67: 11.4% [SD 2.9]; mean MC: 2.3 [SD 1.9]), with necrosis in 27.3% of cases. Carcinoid/NET G3 group (*N* = 23) showed the highest proliferation (mean Ki-67: 25.1% [SD 6.8]; mean MC: 5.5 per 2 mm² [SD 3.3]) and frequent necrosis (60.9%). Cluster structure is depicted in Fig. 2 (upper plots).

**Table 2:** Cluster’ centers using the KAMILA algorithm (k=3) for the variable of interest and comparison with the corresponding WHO classification

	NET G1(N = 383)	NET G2(N = 77)	NET G3(N = 23)
Ki-67 (%)			
Mean (SD)	2.53 (1.69)	11.35 (2.93)	25.09 (6.82)
Median [Q1, Q3]	2.00 [1, 4]	10.00 [9, 14]	25.00 [20, 28]
Min - Max	0–7.00	7.00–18.00	18.00–45.00
Mitotic count (per 2 mm^2^)			
Mean (SD)	0.69 (0.84)	2.34 (1.90)	5.52 (3.34)
Median [Q1, Q3]	1.00 [0, 1]	2.00 [1, 3]	5.00 [3.50, 7]
Min - Max	0–5	0–8	1.00–16
Necrosis			
No, no. (%)	352 (91.9%)	56 (72.7%)	9 (39.1%)
Yes, no. (%)	31 (8.1%)	21 (27.3%)	14 (60.9%)
WHO carcinoid classification			
AC, no. (%)	54 (14.1%)	49 (63.6%)	22 (95.7%)
TC, no. (%)	329 (85.9%)	28 (36.4%)	1 (4.3%)

Abbreviations: no number, *AC* atypical carcinoid, *TC* typical carcinoid

According to the current WHO classification, 125 tumors (25.9%) were ACs and 358 (74.1%) TCs (Fig. 2, bottom plots). TCs were concentrated in Carcinoid/NET G1 (91.9%), with 0.3% and 7.8% assigned to Carcinoid/NET G3 and Carcinoid/NET G2 groups, respectively. By contrast, ACs were distributed across all three Carcinoid/NET clusters, with 43.2% in G1, 39.2% in G2, and 17.6% in G3. Thus, Carcinoid/NET G3 captured almost exclusively a subset of WHO ACs (Table 2), whereas Carcinoid/NET G1 largely overlapped with TCs in terms of cluster composition, albeit including a minority of ACs; notably, Carcinoid/NET G1 still accounted for the largest share of the overall AC cohort. Conversely, Carcinoid/NET G2 comprised a case-mix of both ACs and TCs. In the two-cluster solution, nearly 60% of ACs were grouped with most TCs within the low-proliferation cluster, underscoring the imperfect correspondence between necrosis- and mitosis-based WHO criteria and the proliferative landscape captured by clustering (see Supplementary Results 1, Section c; Supplementary Fig. 2; Supplementary Table 3). Overall, Ki-67 emerged as the most powerful discriminator, followed by MC, while necrosis was negligible, suggesting potential value in prioritizing quantitative proliferative indices (Ki-67 and MC) over the binary necrosis assessment.

### Prognostic Value of the Obtained Data-Driven Classification

**Fig. 3:**
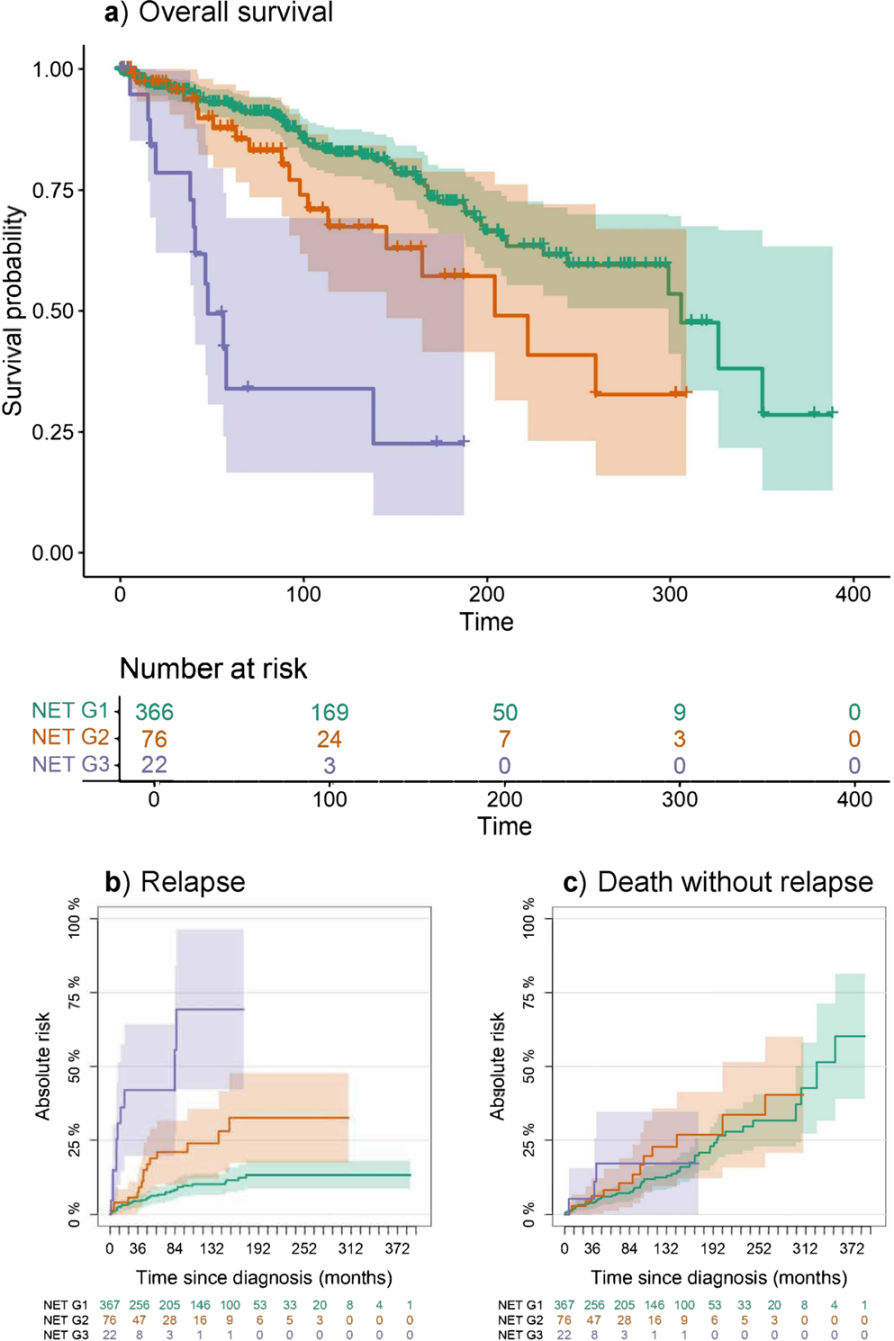
Kaplan-Meier (KM) survival curves (**a**) and Aalen-Johansen (AJ) crude incidence estimates of relapse (**b**) and death without relapse (**c**) by the 3-cluster solution (NET G1, NET G2, NET G3). Censored observations are marked with “+” along the curves. Tables report the number at risk for the corresponding event, that is how many patients in each group remain under observation at successive time points

In a univariable Cox model with Carcinoid/NET G2 as the reference, the HR for death was 3.98 (95% CI: 1.91–8.29) for Carcinoid/NET G3 and 0.57 (95% CI: 0.34–0.95) for Carcinoid/NET G1; proportional hazards assumption was not violated (global Schoenfeld test χ²(2) = 0.99, p-value = 0.61). KM curves showed a clear prognostic gradient with increasing unfavorable life expectancy from Carcinoid/NET G1 to G3 groups (Fig. 3A; see Supplementary Results 1, Section d). Carcinoid/NET G2 appeared as an intermediate-risk stratum, clearly separated from G3, closer to G1 within 5 years, but with larger separation at longer follow-up. For comparison, Supplementary Fig. 3 shows the KM curves estimated for patients grouped according to the current WHO carcinoid classification. WHO classification and the obtained groups showed similar prognostic discrimination in the univariable model (p-value = 0.319; see Supplementary Results 1, section d), but model fit favored clusters (AIC = 1023.7) over WHO (AIC = 1030.8).

In a multivariable Cox model including gender and age (both significant in univariable models), adding cluster information improved fit *versus* a model with age and gender only (likelihood ratio test [LRT], χ²(2) = 10.05, p-value = 0.007; Supplementary Table 5). However, comparison of multivariable models including gender and age with either clusters alone or clusters and WHO classification showed no significant difference in model fit (LRT, χ²(1) = 2.36, p-value = 0.125), possibly due to shared classification criteria, as well as the detrimental effect of age and male gender on NET prognosis.

Carcinoid/NET G3 showed 10 relapses and 3 deaths before relapse; G2 showed 15 relapses and 13 deaths before relapse; and G1 had 33 relapses and 56 deaths before relapse. The cumulative incidence of recurrence (Fig. 3B) differed across clusters (Gray test χ²(2) = 43.719, p-value = 3.210⋅10^⁻10^), whereas death without recurrence (Fig. 3C) did not (χ²(2) = 1.12, p-value = 0.570), indicating the role of competing factors. Recurrence incidence was highest in Carcinoid/NET G3, lowest in G1, and intermediate in G2. The latter occupied an intermediate position: its recurrence risk was clearly lower than G3 and closer to G1, particularly in the earlier follow-up, while the absence of differences in death without prior recurrence indicates that the separation in disease-free outcomes was driven by recurrence rather than differential non-recurrence mortality.

## Thresholds

**Fig. 4:**
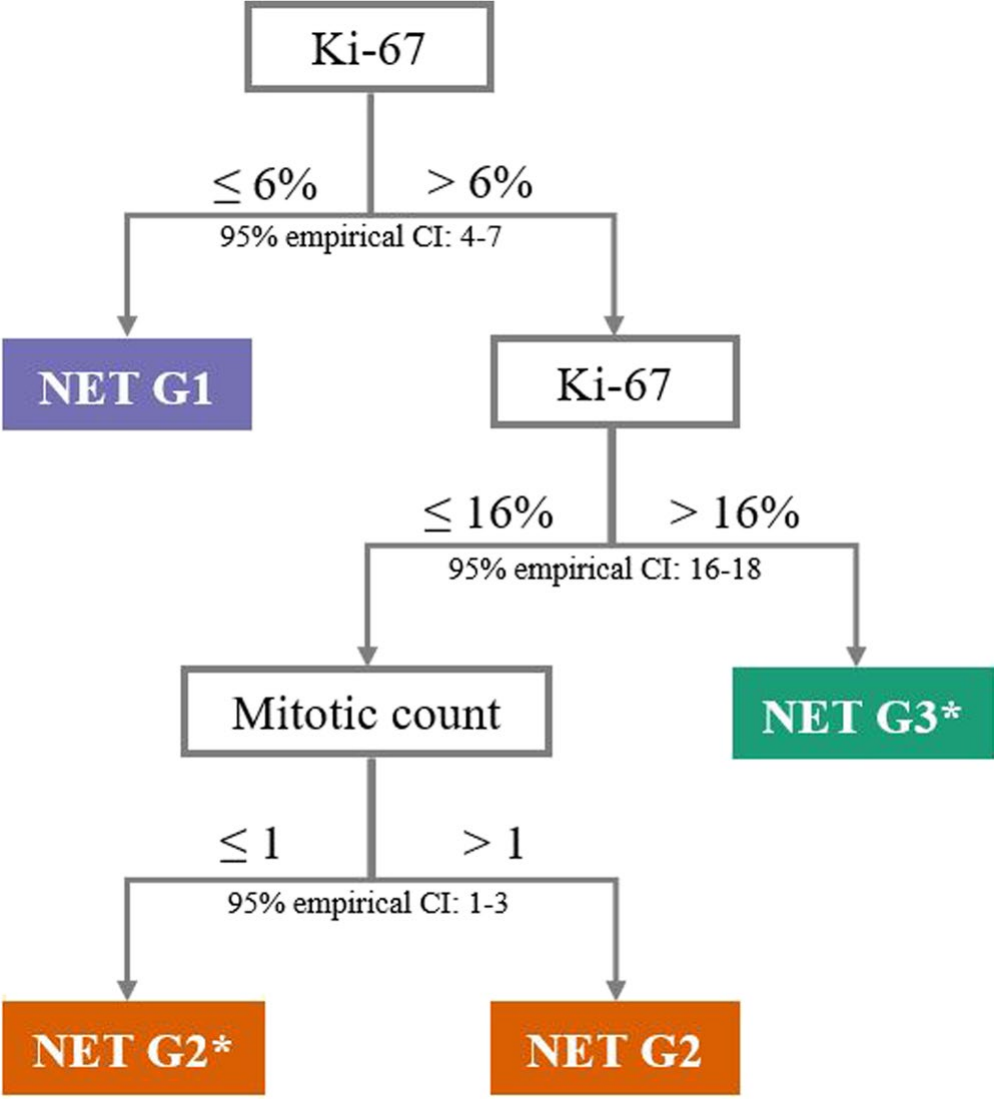
Suggested classification system assigning cases to the data-driven clusters (NET G1, NET G2, and NET G3) using Ki-67 and mitotic count. The tree splits on Ki-67 (≤ 6%; >16%) and, in the intermediate range (6%< Ki-67 ≤ 16%), on mitotic count (≤ 1 vs. > 1). The 95% empirical confidence intervals (CIs) indicate uncertainty ranges for the cut-offs. Each leaf (end of a path) reports the suggested group. * indicates mixed classifications

The derived decision tree (Fig. 4) used Ki-67 as the primary discriminant and the MC in the intermediate range. For Ki-67 ≤ 6% (95% empirical CI: 4–7%), all cases falling into this threshold-defined group had originally been assigned to Carcinoid/NET G1 by clustering. In cases with Ki-67 > 16%, the corresponding threshold-defined group included all Carcinoid/NET G3 and two cases originally assigned to Carcinoid/NET G2 by clustering (both showing Ki-67 = 18%). In the intermediate range of 6% < Ki-67 ≤ 16% (95% empirical CI: 16–18), the large majority (81.6%) of cases with MC ≤ 1 (95% empirical CI: 1–3) belonged to the Carcinoid/NET G2 cluster, while the remaining 18.4% had originally been assigned to Carcinoid/NET G1. Within the same Ki-67 range, cases displaying MC > 1 were all originally classified as Carcinoid/NET G2.

These findings suggest that Ki-67 effectively discriminates between low- and high-proliferation groups, while, in the intermediate “gray zone”, it could require integration of MC to achieve more accurate classification, displaying two further subsets of Carcinoid/NET G2. Of note, it was Ki-67 that ultimately drove the final grading, as these Carcinoid/NET G2 tumor subsets converged on the same prognostic assignment, whereas necrosis did not contribute at all to grading (see Supplementary Results 1, Section f).

## External Cohort (Supplementary Results 1, Section g)

**Fig. 5:**
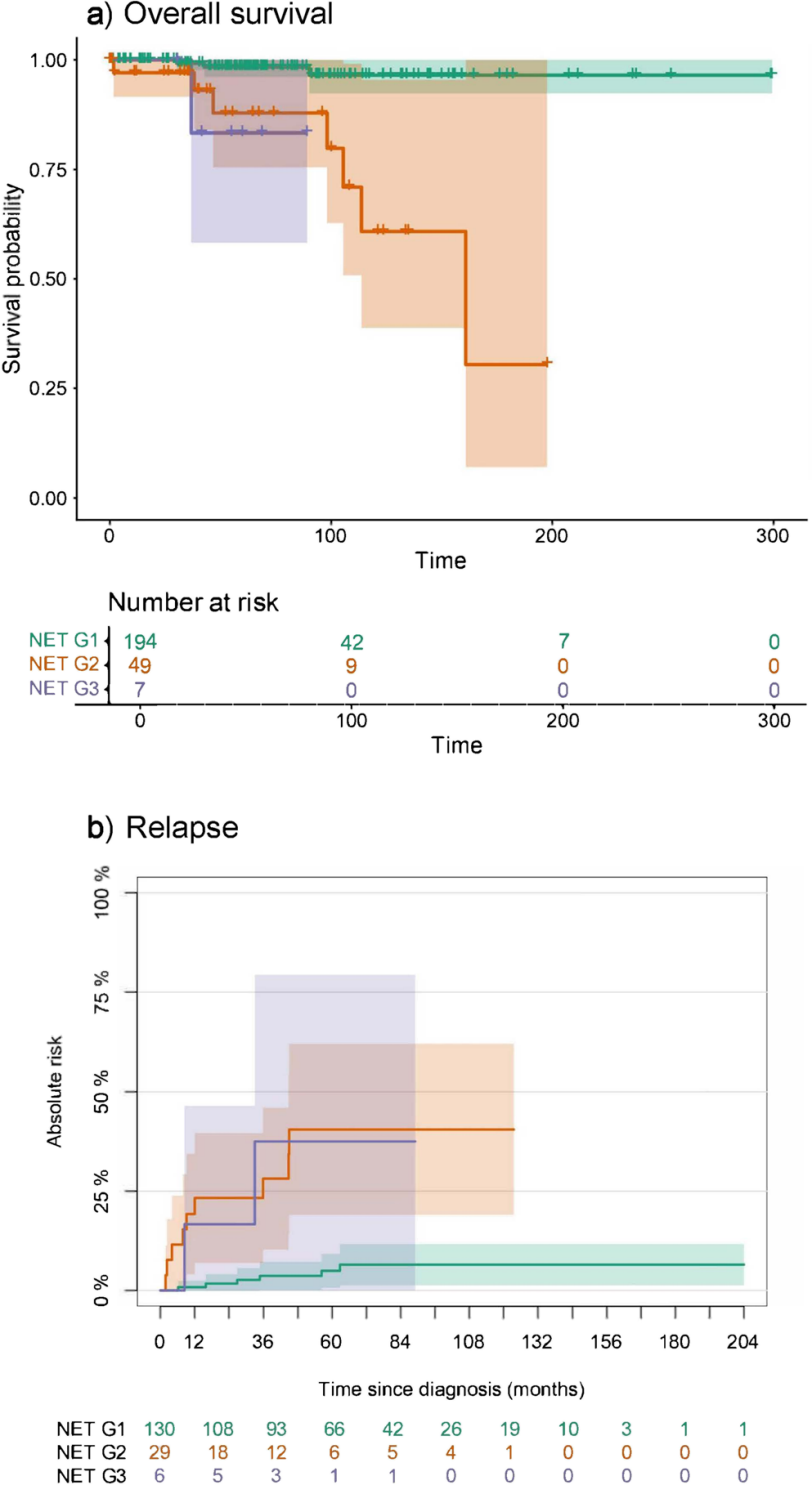
Kaplan-Meier (KM) survival curves (**a**) and Aalen-Johansen (AJ) crude incidence estimates of relapse (**b**) for observations from the external cohort, classified according to our proposed classification (NET G1, NET G2, NET G3). Censored observations are marked with “+” along the curves. Table reports the number at risk for the corresponding event, that is how many patients in each group remain under observation at successive time points

The external validation cohort of 259 cases (of which 253 were usable) was analyzed according to the same statistical procedures, by firstly applying the algorithm to delineate three groups, and by secondly introducing the predefined cut-off thresholds for Ki-67, because MC and necrosis resulted in less effective and even irrelevant outcomes for prognostic stratification, respectively. Observations from the external cohort assigned to clusters based on the hard classification are shown in Supplementary Fig. 5. Subsequently, carcinoids were classified using Ki-67 thresholds alone: 197 tumors were classified as Carcinoid/NET G1 (of them 33 were part of Carcinoid/NET G2), 49 as Carcinoid/NET G2 (of which 20 were in Carcinoid/NET G3), and 7 as Carcinoid/NET G3 (Supplementary Fig. 4, bottom); the adjusted rand index between the manual and the clustering partition was 0.6. Within each group, 4, 7, and 1 deaths occurred, respectively. KM curves for OS (Fig. 5A) differed between the Carcinoid/NET groups (overall p-value = 8⋅10^− 5^). In particular, G1 differed from both G2 and G3 survival rates (G1 *versus* G2 p-value = 3⋅10^− 5^; G1 *versus* G3 p-value = 0.032). G2 and G3 curves of behaviors did not significantly differ (p-value = 0.721). Similarly, crude incidence curves (Fig. 5B) overall differed between the groups (p-value = 4.65⋅10^− 6^), particularly between Carcinoid/NET G1 and both G2 and G3 (G1 *versus* Carcinoid/NET G2 p-value = 5.14⋅10^− 6^; G1 *versus* Carcinoid/NET G3 p-value = 4.67⋅10^− 3^), but not between Carcinoid/NET G2 and G3 (p-value = 0.978). However, it should be noted that G3 cases were under-represented in the external cohort.

## Discussion

In this study, we propose an innovative prognostic grading system for pulmonary Carcinoid/NETs based on the combination of essential WHO criteria (necrosis and MC) and the crucial addition of Ki-67, a marker currently considered desirable. Moreover, the WHO classification applies solely to resection specimens, whereas the descriptive terms Carcinoid/NET NOS and metastatic carcinoid NOS have been introduced for small diagnostic samples based on distinct clinical contexts. Moreover, the provisional category of proliferative carcinoids has been described as well, but no precise relationship to tumor behavior has been provided in the dichotomous subdivision of TC and AC. Another issue is that necrosis and mitoses are difficult to assess in small tissue samples, whereas Ki-67 provides a rapid estimate of cell proliferation in any clinical context. Of note, each carcinoid category, whether TC or AC, is clinically, behaviorally, and molecularly heterogeneous, but these differences are not satisfactorily captured by the current WHO classification [5, 8, 42, 43]. Our study, exclusively focused on NETs, allowed the identification of three prognostic tiers where Ki-67 is the key player of classification, highlighting Carcinoid/NET G1, G2 and G3, whereas MC helps identify only a seven-case subset of G2 tumors characterized by lower proliferation (< 1 mitosis and 6–16% Ki-67), which was not prognostically relevant. Notably, Carcinoid/NET G1 also included some ACs, even with necrosis, underscoring that necrosis alone does not fully capture carcinoid aggressiveness. This finding is only seemingly at odds with the established defining role of necrosis, which, although observed in no more than 10–15% of pulmonary carcinoids and subjected to a non-marginal interobserver variability [9, 27], constitutes a traditional criterion for AC [44]. Nonetheless, recent studies have not identified necrosis as an independent prognostic factor in Carcinoid/NETs, once proliferative activity is accounted for [45] which aligns with the substantial co-segregation of necrosis with higher Ki-67 and MC we observed in this study. However, a subset of carcinoids with necrosis (i.e., AC by WHO definition) exhibited a Ki-67 index < 6% and a favorable prognosis, in keeping with studies where necrosis may be detected even in low proliferating Carcinoid/NETs [5, 46–48]. An explanation could be that necrosis may arise independently of high proliferative activity, due to ischemic or hypoxic microenvironment in larger tumor aggregates, immune-mediated reactions, intratumoral hemorrhage, or degenerative changes. Accordingly, in our model, necrosis was insufficient for a robust prognostic classification, if not in conjunction with MC and Ki-67. Our data suggests that necrosis is not an independent prognostic parameter distinct from proliferative activity, with which it appears highly colinear. Therefore, when necrosis is associated with low-to-intermediate proliferative activity, as observed in carcinoids/NETs G1 and G2, it does not support a prognostic role on its own. However, to make this comparison possible, it is necessary to assess and describe necrosis, also because pulmonary pathologists are familiar with punctate necrosis in lung NETs, regardless of its underlying mechanisms of formation that could explain its different biological role. Accordingly, necrosis could become a desirable but not essential criterion in the prognostic stratification of pulmonary NETs, which would merit further investigation in dedicated studies.

The mixed composition of the three clusters demonstrated that this classification is largely independent of histological subtyping in surgical specimens and has the potential to be applicable to biopsies and cytology samples of primary and metastatic sites as well, as it primarily relies on Ki-67 rather than MC and necrosis, both of which may miss recognition in such contexts. The combination of Ki-67 and MC to grade Carcinoid/NETs implies that these factors are independent and synergistic. In routine practice, the evaluation of mitotic activity across hematoxylin and eosin-stained slides remains a key morphological step, as it guides the selection of the most appropriate tumor areas for subsequent Ki-67 hotspot assessment. Consequently, if one of the two parameters is unavailable (e.g., mitoses in small biopsies or Ki-67 in limited-resource settings), the system still allows the assignment of a risk category.

The selected cut-offs in our study were derived from a statistical approach evaluating hundreds of decisional-tree configurations, following a logical sequence from Ki-67 to MC. A large body of literature on Ki-67 reports 5% as a widely agreed-upon expert consensus threshold to separate TC and AC or even a prognostic factor for more aggressive TCs [49], while 20% is used to define proliferating carcinoids [4].

While the WHO classification has proposed a Ki-67 cut-off > 30% for defining proliferating carcinoids, in agreement with expert reviews and studies supporting this threshold [5, 50–53], other evidence suggests that a lower cut-off of 20% may be sufficient to identify these proliferating NETs. This threshold appears to be more closely aligned with the carcinoid/NET G3 category we observed in our series [46, 54, 55]. These figures are quite close to our observed values of 6% (95% empirical CI: 4–7%) and 16% (95% empirical CI: 16–18%), which confirm their potential for prognostic stratification. Interestingly, while the dichotomous WHO classification and the unsupervised three-tier clustering performed similarly as standalone predictors of OS, the added value of our Ki-67-based model provided meaningful sub-stratification, particularly within AC, which is the most unpredictable category of carcinoids, and where NET G2 behaved similarly to NET G1 subsets but distinctly from higher-risk NET G3. This observation further underscores the importance of integrating Ki-67 and MC for classifying intermediate-proliferating NET G2, whereas necrosis was irrelevant even in the NET G1 category. In this context, our model outperformed the WHO classification of TC and AC, which does not adequately capture the inherent heterogeneity of each tumor category, despite efforts to identify MC and Ki-67 in diversely behaving AC [44]. In our view, three prognostic groups better described tumor behavior than two, as ACs were segregated into better-behaving tumors assigned to Carcinoid/NET G1 and more aggressive tumors assigned to Carcinoid/NET G3 based on Ki-67, and identified a true intermediate category through the synergistic contribution of Ki-67 and MC. Moreover, the same model revealed the aggressiveness of rare TCs, which clustered with Carcinoid/NET G2 and even Carcinoid/NET G3.

In our study, we chose to retain the widely known term “carcinoid” in the lung to denote well-differentiated tumors, while adding the suffix NET G1 through G3 to stratify their malignancy. The terminology Carcinoid/NET G1–G3 is used here to reflect a three-tier stratification of pulmonary carcinoids derived from our analysis. Although similar terminology is used in other neuroendocrine neoplasms, the cut-offs identified in this study are specific to pulmonary carcinoids and are not intended to reproduce the grading systems used in gastro-enteropancreatic NETs. The Carcinoid/NET G3 category, although formally not including highly proliferative carcinoids according to the 2021 WHO’s definition, was nevertheless consistent with literature data regarding Ki-67 > 20% [4, 16, 28], identifying more aggressive tumors than Carcinoid/NET G2, analogous to the GEP NET G3 category. In lung pathology, the term “Grade 3” is traditionally reserved for poorly differentiated neuroendocrine carcinomas (LCNEC and SCLC). In the present study, the Carcinoid/NET G3 category instead refers to well-differentiated tumors with higher proliferative activity and should not be interpreted as equivalent to poorly differentiated NECs, but rather as a proliferative stratification within well-differentiated pulmonary carcinoids.

In line with this perspective, recent work has proposed a GEP-NET–like grading system for pulmonary NETs based solely on Ki-67, supporting the adoption of a three-tier (G1–G3) classification. While our findings are consistent with the prognostic relevance of Ki-67, our data further suggest that integrating Ki-67 with mitotic count provides a more refined stratification, particularly within intermediate proliferative ranges [56].

A potential alignment between the proposed 3-tier system and known molecular subtypes of pulmonary NETs should also be considered in the paper for the sake of clarity and completeness of interpretation. Distinct profiles defined by markers such as OTP, ASCL1, and CD44 have been associated with differences in tumor biological properties and clinical behavior [8, 11, 22, 42, 57]. Although molecular data were not available in our study, the observed stratification mainly based on proliferative activity may reflect underlying molecular heterogeneity of tumors. Low-proliferation tumors (carcinoid/NET G1) may correspond to more differentiated and less aggressive subtypes (e.g., OTP-positive, ASCL1 negative, CD44 negative), whereas higher-proliferation groups may overlap with more aggressive tumor profiles (e.g., OTP-negative, and ASCL1- or CD44-associated). Although plausible, this remains a speculative interpretation due to the lack of molecular data, whereby representing a limitation of the present study. Further research integrating histopathologic and molecular findings is therefore warranted to expand the biological insights of our study.

The predictive value of our three-tier model seemed to be extendable to OS and it was particularly significant for RFS, which more directly reflects the underlying biological profile of the disease. In multivariable analysis, using Carcinoid/NET G2 as the reference, the Carcinoid/NET G1 group emerged as an independent predictor of favorable prognosis. This is unsurprising, insofar it predominantly consists of TC, though included a non-negligible number of ACs, some of which even with necrosis that did not nonetheless impact survival. Carcinoid/NET G3 was not independent in multivariable analysis, but the confidence intervals were wide enough to suggest a potential prognostic role, probably owing to their small number in this category.

A strength of our study lies in the large sample size and extended follow-up, surpassing most published series, as well as the use of standardized immunohistochemistry and slide review by dedicated pulmonary pathologists (MV, LR, GP, MP). However, the analysis was performed exclusively on resected primary pulmonary carcinoids, and the proposed stratification model has therefore not been formally validated in metastatic lesions or small biopsy specimens. Although Ki-67 assessment can be performed on limited tissue samples, its evaluation in small biopsies may be influenced by tumor heterogeneity and hotspot selection, and further studies will be necessary to assess the reproducibility and clinical applicability of this approach in non-resection material. Randomized assignment for pathological assessment further minimized inter-observer variability in evaluating mitoses, necrosis, and Ki-67, although a formal reproducibility study was not conducted. Even Carcinoid/NET G1, expected to have the best prognosis, showed progressive OS decline over time, likely due to competing factors such as age and comorbidities (mean age 63 years), as also confirmed by the multivariable model. A similar trend was observed in WHO-classified TC, which, despite their generally favorable prognosis, also exhibited OS deterioration for the same reasons. In this context, RFS analysis showed significantly fewer recurrences in Carcinoid/NET G1 than G2, and fewer in G2 than G3, supporting the accuracy of our three-tier model when recurrence is considered as the clinical endpoint. This highlights an underrecognized point in the literature: OS declines continuously beyond the usual 10–20 years owing to competing events, whereas RFS provides greater clinical value for establishing grading systems, inasmuch as it is more directly influenced by tumor behavior. Another key strength of our study was the external cohort of 253 NETs, even if only Ki-67, MC, necrosis, and survival data were available for statistical analysis. Despite differences in tumor composition, with fewer high-Ki-67 cases, both the proposed classification and the new clustering performed even better than rigid cut-offs, highlighting its ability to generalize a three-tier grading in lung NETs across independent cohorts, and adapt to variable pathological distributions. As a matter of fact, this clustering approach, looking for the interrelations of mitoses, necrosis, and Ki-67 and employing a decisional tree methodology, represents an unprecedented statistical method applied to the prognostic stratification of lung NENs. However, validating the classification system in terms of survival outcomes in the external cohort was limited by the small number of G3 cases. Therefore, future work should focus on validating the classification in larger cohorts.

In conclusion, the terminology Carcinoid/NET G1 through G3 is proposed herein for comprehensively classifying lung NETs. It has been developed on resection specimens but may potentially be adapted to biopsy material, as hot-spot-based Ki-67 is the key player of classification. It is independent of TC and AC subtyping, applicable to carcinoids with high proliferation rates as well, and may contribute as a proposal for future classifications and clinical studies.

## Supplementary Information

Below is the link to the electronic supplementary material.

Supplementary Material 1

## Data Availability

No datasets were generated or analysed during the current study.
