# Supplementary material for: Three-Tier Prognostic Stratification of Lung Carcinoids (NET G1-G2-G3) by Multivariable, Data-Driven Integration of Ki-67 and Mitotic Count
Source: Endocr Pathol. 2026 May 21;37(1):24. doi: 10.1007/s12022-026-09920-4 (PMC13194188; doi:10.1007/s12022-026-09920-4)
Supplement: Supplementary file 1 — Supplementary Material 1 [file 12022_2026_9920_MOESM1_ESM.docx]

**Three-tier prognostic stratification of lung carcinoids (NET G1-G2-G3) by multivariable, data-driven integration of Ki-67 and mitotic count**

Giulia Orlando^1^*, Valentina Veronesi^2^*, Eleonora Duregon^1^, Vanessa Zambelli^3^, Francesco Leo^4^, Elisa Carla Fontana^5^, Enrico Ruffini^5^, Luisella Righi^3^, Giuseppe Pelosi^6^, Marco Volante^3^, Mauro Papotti^1^

^1^Division of Pathology, City of Health and Sciences University Hospital, Department of Oncology, University of Turin, Turin, Italy.

^2^Department of Biomedical, Surgical and Dental Sciences, University of Milan, Milan, Italy.

^3^Division of Pathology, San Luigi University Hospital, Department of Oncology, University of Turin, Orbassano, Italy.

^4^Division of Thoracic Surgery, San Luigi University Hospital, University of Turin, Orbassano, Italy.

^5^Division of Thoracic Surgery, City of Health and Sciences University Hospital, University of Turin, Turin, Italy.

^6^Department of Oncology and Hemato-Oncology, University of Milan, Milan, Italy.

*Equally contributed as first authors

**CORRESPONDING AUTHORS**

Prof M. Volante, Division of Pathology, San Luigi University hospital, Regione Gonzole 10, 10043 Orbassano, Torino, Italy - Email: marco.volante@unito.it

and Prof G. Pelosi, Inter-Hospital Division of Pathology, IRCCS MultiMedica, Via Gaudenzio Fantoli, 16/15, 20138 Milan, Italy – Email: giuseppe.pelosi@unimi.it

**SUPPLEMENTARY METHODS**

***Supplementary Methods, Section a) Immunohistochemistry.*** After revision of hematoxylin and eosin (H&E) slides and immunohistochemistry for neuroendocrine markers performed at the time of diagnosis (Chromogranin A, Synaptophysin, INSM1), representative tissue blocks were selected for Ki-67 staining. Immunohistochemistry was performed on two automated platforms: BenchMark ULTRA AutoStainer (Ventana Medical Systems, Tucson, AZ, USA) using the Ki-67 rabbit monoclonal primary antibody (clone 30-9, prediluted, Ventana Medical Systems, Tucson, AZ, USA), and Dako Omnis (Agilent, Santa Clara, CA, USA) using the Ki-67 mouse monoclonal primary antibody (clone MIB-1, prediluted, Agilent, Santa Clara, CA, USA). Appropriate positive and negative controls were included in each immunohistochemical run. Ki-67 was calculated as the percentage of Ki-67 positive nuclei in 2000 tumor cells that were counted in areas with the highest labeling (“hot spots”).

***Supplementary Methods, Section b) Statistical analysis.*** Cluster analysis is an unsupervised, exploratory technique that aims at partitioning collection of observations into homogeneous groups, *i.e.*, clusters, so that objects within the same cluster are more similar to each other than to those in different clusters [1]. Specifically, we used the KAMILA (KAy-means for MIxed LArge data) algorithm [2], that is specifically designed for mixed-type data (*i.e.*, data that comprise realizations of different variable types), to cluster observations using necrosis (categorical), mitotic count (MC), and Ki-67. We examined solutions with k=2 clusters to test whether an unsupervised, data-driven partition would reproduce the TC/AC separation, and with k=3 clusters to probe a potential intermediate group. The intuition of using k=3 was based on expert knowledge and its selection was validated using the Calinski-Harabasz (CH; the higher the better) and the Silhouette (the higher the better) indexes. Necrosis was casted into a 0-1 interval/ratio scale variable; number of clusters k=2 to k=7 were compared. The CH index, measuring the ratio of the between-to-within cluster dispersion, suggested k=3 (for k=3: CH=1025 vs. CH ≤ 940). While the Silhouette index, measuring the “correctness” of assignments, suggested comparable results for k=2 (Silhouette=0.60) and k=3 (Silhouette=0.52), and lower values for k≥4 (Silhouette≤0.38). MC and Ki-67 were modeled as interval/ratio scale (continuous) variables; because MC is discrete in nature, we repeated the analysis with categorized MC, which changed assignments for only 10 observations, whereby retaining the continuous specification. KAMILA was implemented with 10 random starts using the kamila R package (version 0.1.2) [3], and number of clusters as 2 or 3 dependently on the case, while all the other parameters were left at their defaults. Ki-67 was not assessable in one case and was treated as missing and addressed with multiple imputations. We used the mice R package (version 3.16.0) to generate five plausible values for Ki-67 by chained equations with predictive mean matching, using gender, age, MC, necrosis, tumorlets, multifocality and tumour location as predictors. Cluster analysis was run on the five imputed dataset, and the final labeling was obtained by majority voting. Furthermore, a sensitivity analysis was conducted to evaluate the stability of the partition when the patient with the not assessable observation was excluded. As there was perfect agreement, results are not presented.

To assess whether the data-driven three-cluster solution carried prognostic information for overall survival (OS) and to benchmark it against the WHO TC/AC classification, we ran survival analyses restricted to patients with complete status information and last follow-up/death dates. Follow-up was summarized by reverse Kaplan-Meier (KM). OS was estimated with KM curves, with global and pairwise log-rank tests (Holm adjustment) and point estimates at 12, 36, and 60 months. We computed restricted mean survival time (RMST) truncated at 60 months and compared groups via pairwise RMST differences (Holm-adjusted). Prognostic effects were quantified using Cox proportional hazards models: univariable models with cluster (or WHO class) and multivariable models additionally adjusting for age and gender. Finally, we evaluated added value beyond WHO by fitting Cox models within WHO strata (TC and AC) including the cluster labels. The proportional hazards assumption was checked with Schoenfeld residuals. Predictive performance was compared using Harrell’s C-index with optimism correction via 1000 bootstrap resamples, Akaike Information Criterion (AIC), and 60-month time-dependent AUC and Brier score. Crude incidence curves of relapse free survival (RFS) and death as competing events were calculated using the Aalen-Johansen (AJ) estimator. The Gray test was used to compare incidence curves between clusters.

To translate the data-driven clusters into clinically usable thresholds, we fitted conditional inference trees [4] to predict cluster labels from Ki-67 (%), MC, and necrosis. Conditional inference trees were trained, using the ctree function in partykit R package (package version 1.2.24) [4], to predict cluster labels from Ki-67 (%), MC, and necrosis. We performed 5-fold cross-validation repeated 100 times (500 trees total) and quantified (i) reassignment accuracy, the proportion of cases whose tree-assigned label matched the original cluster label, and (ii) cut-point stability, frequency with which each predictor was selected at internal nodes and the empirical distribution of its split values. Misclassification patterns were summarized via cross-validated confusion matrices, and variable-usage counts provided an important summary. The resulting tree-based rule was obtained by fixing the split thresholds at consensus cut-points obtained across resamples (majority voting); terminal nodes were assigned by majority cluster and annotated with their empirical cluster proportions. Around each consensus cut-point, we considered the empirical 95% interval of the resamples split values in a local neighborhood of the cut-point (±3 for Ki-67% and ±2 for MC); if less than 10 splits fell in the window, the 25 nearest splits were used.

An external cohort (N=259) was used to replicate the clustering [5]. Data were freely available at in the ESMOOpen_LungNENomicsCohort repository (<https://github.com/IARCbioinfo/ESMOOpen_LungNENomicsCohort>). In the external cohort, for each carcinoid, up to six measurements performed by six different pathologists, were available for Ki-67 index, MC, and necrosis. For necrosis and MC, we summarized the measurements into a single value using the median and excluding missing values. Necrosis presence was defined by majority voting. In two cases where there was an equal proportion of votes, the final necrosis status was decided based on Ki-67: absence in the case with low Ki-67 value, and present in that with higher Ki-67 value. Six cases were excluded because Ki-67 measurements were not recorded by any of the pathologists. After data processing, 253 observations were retained for the analysis. To group the observations from the external cohort, we re-ran clustering *ex novo* with k=3, using the same procedure as for our test sample. We then assigned patients based on the *hard* (i.e., without considering the 95% empirical CI) thresholds in the classification tree we obtained from our cohort. In cases of multiple possible classifications, the carcinoid was assigned to a class based on the higher of the two values considered for the decision. The Adjusted Rand Index (ARI), a measure of agreement between two groupings, was used to compare clustering partition and manual classification. The ARI is upper bounded by 1; the higher the ARI, the more concordant the partitions. OS and RFS were then compared across groups via KM curves and log-rank tests, and AJ estimator and Grey tests, respectively; p-values for multiple comparisons were adjusted using the Holm method. Among patients with complete information in Ki-67%, MC, and necrosis presence, RFS data were only available for 166 of them; the remaining were excluded from RFS analysis.

**SUPPLEMENTARY RESULTS**

**Supplementary Results 1, Section a). Supplementary Fig. 1** showed that nearly all tumors without necrosis clustered in the lower-left quadrant of the Ki-67- MC space, with Ki-67 ≤10% and MC <2 per 2 mm². In contrast, necrosis-positive tumors shifted upward and rightward, occupying regions with both higher Ki-67 and MC, although a small subset with necrosis remained in the “low-low” corner. The most frequent combination overall was 1% Ki-67 with 0 mitoses and no necrosis, and the density of cases decreased steeply once Ki-67 exceeded 5% or mitoses exceeded 1. A diagonal trend was suggested whereby MC increased in parallel with Ki-67 values once Ki-67 ≥4%, with necrosis-positive cases populating the higher end of this diagonal. Very few tumors showed extremely high values on both axes (e.g., Ki-67 >20% and >8 mitoses).

**Supplementary Results 1, Section b)**. The WMW test, comparing Ki-67 and MC distributions between necrosis positive vs. negative carcinoids, suggested that both Ki-67 index and MC had higher values in the necrosis-positive group (Ki-67: W = 7256, p < 0.001; mitoses: W = 5497.5, p < 0.001). The estimated median shift indicated that tumors with necrosis had on average a 4% higher Ki-67 index (95% CI: 2–7) and 2 additional mitoses per 2 mm² (95% CI: 1–2) compared with necrosis-negative tumors. Effect size estimates suggested the same difference direction: Cliff’s δ was 0.47 (95% CI: 0.35–0.58) for Ki-67, indicating a medium-to-large effect, and 0.60 (95% CI: 0.50–0.69) for MC, indicating a large effect. In other words, there was a 47% excess probability that a necrosis-positive tumor would have a higher Ki-67 than a necrosis-negative one, and a 60% excess probability of having more mitoses.

**Supplementary Results 1, Section c)**. When seeking for k=2 clusters (**Supplementary Fig. 3**; **Supplementary Table 5**), *Cluster 2* (N=60) represented the more proliferative group, with a mean Ki-67 of 18.2% (SD 7.1) and a mean MC of 4.1 per 2 mm² (SD 2.8). Necrosis was present in nearly half of the cases (48.3%). *Cluster 1* (N=423) contained the majority of tumors, with much lower proliferation (mean Ki-67 3.1%, SD 2.5; mean MC 0.8, SD 0.9), absence of necrosis in 91.3%, and only a minority showing necrosis (8.7%). In terms of WHO classification, TCs were almost entirely assigned to *Cluster 1* (97.8%), whereas ACs were split between the two clusters (58.4% in *Cluster 1*, 41.6% in *Cluster 2*).

**Supplementary Results 1, Section d)**. Regarding OS, 19 patients were excluded due to missing status and last follow-up dates, leaving 22, 76, and 366 patients in Carcinoid/NET G3, Carcinoid/NET G2, and Carcinoid/NET G1, respectively. Deaths were 12/22 (54.5%), 19/76 (25.0%), and 70/366 (19.1%), with censoring proportions of 45.5%, 75.0%, and 80.9%, respectively. Median follow-up by reverse KM was 110.4 months overall, and 69.6, 83.2, and 115.0 months for Carcinoid/NET G3, G2 and G1 groups, respectively. The Carcinoid/NET G3 curve lies below Carcinoid/NET G2 and Carcinoid/NET G1 groups throughout follow-up, while Carcinoid/NET G2 is intermediate and overlaps Carcinoid/NET G1 early in follow-up. At 60 months, overall survival was 34% (95% CI: 17-69%) in Carcinoid/NET G3, 88% (95% CI: 80-97%) in Carcinoid/NET G2, and 92% (95% CI: 90-96%) in Carcinoid/NET G1. Survival at 12 and 36 months is reported in **Supplementary Table 4**. A global log-rank test indicated differences in survival among clusters (χ²(2)=48.3, p-value=3⋅10⁻¹¹); Holm-adjusted pairwise log-rank p-values were 1.6⋅10⁻⁴ (Carcinoid/NET G3 vs Carcinoid/NET G2), 7.8⋅10⁻¹¹ (Carcinoid/NET G3 vs Carcinoid/NET G1), and 0.030 (Carcinoid/NET G2 vs Carcinoid/NET G1). Using a 60-month horizon, the RMST was 44.7 months for Carcinoid/NET G3, 56.6 months for Carcinoid/NET G2, and 57.4 months for Carcinoid/NET G1. Pairwise RMST differences (second minus first group) were: Carcinoid/NET G2 vs Carcinoid/NET G3, 11.9 months (Holm-adjusted p-value=0.006); Carcinoid/NET G1 vs Carcinoid/NET G3, 12.7 months (p-value=0.002); Carcinoid/NET G1 vs Carcinoid/NET G2, 0.8 months (p-value=0.577). Discrimination of the Cox model including the obtained clusters only was moderate (Harrell’s C-index: 0.60 [95% CI 0.54-0.65]; optimism-corrected C-index with 1000 bootstraps: 0.59 [95% CI: 0.54-0.65]). In other words, the ability of the single-predictor-model to rank patients’ mortality risk is limited. In turn, the current WHO classification alone had similar prognostic value (C-index: 0.62, 95% CI: 0.58-0.68), with no significant difference with respect to the obtained clusters (|ΔC|=0.02, p-value=0.319).

**Supplementary Results, Section e)**. Adding Ki-67% to WHO classification as an independent predictor in the model yielded comparable fit to the cluster model (AIC = 1025.6), suggesting that clustering-generated-groups’ gain over current WHO classification was largely attributable to incorporating Ki-67 (and a data-driven partition). The estimated AUC (at 60 months) for the clusters’ model, the current WHO classification, and the combined model (comprising both the clusters and the WHO classification) were 66.5%, 68.0%, and 70.2%, respectively, suggesting some synergistic effect by merging proliferation and histological subtyping, partially attributable to Ki-67 contribution. Overall, clusters and WHO classification performed similarly when tested as standalone predictors for OS; the three-tier solution, mainly linked to Ki-67%, provided sub-stratification particularly within ACs, with Carcinoid/NET G2 behaving closer to Carcinoid/NET G1 but distinct from the higher-risk Carcinoid/NET G3.

The accuracy of probabilistic predictions by means of Brier’s scores, where lower values indicate better overall prediction error, was 8.4% for the clusters’ model, 9.2% (WHO model), and 8.3% (both predictors’ model), with only the “both vs. clusters” models Brier comparison non-significant. We tested also the prognostic value of the clusters within WHO classification’s groups. Within TC, clusters did not further stratify risk (LRT p-value=0.801), whereas within AC it did (LRT p-value=0.005), with higher, non-significant, hazards for Carcinoid/NET G2 (HR=1.37, 95% CI: 0.61-3.07, p-value=0.441) and higher hazard for Carcinoid/NET G3 (HR=4.02, 95% CI: 1.78-9.05, p-value=0.008) compared to Carcinoid/NET G1.

**Supplementary Results, Section f)**. To verify the clinical transferability of the labels obtained from clustering, we estimated 500 conditional trees using repeated 5-fold splits and evaluated their cut stability and reassignment capacity. The average reassignment was 0.991 (SD 0.010; range 0.935–1.000). Residual errors were almost exclusively concentrated between the Carcinoid/NET G3 and Carcinoid/NET G2 groups, while Carcinoid/NET G3 group was classified almost perfectly. Analysis of the use of variables at internal nodes confirms the predominant role of Ki-67, which was selected as splitting variable 1398 times across 500 trees at all levels, compared to MC (456). Necrosis was never selected.

**Supplementary Results, Section g). External cohort.** The external cohort differed from our cohort in terms of the presence of necrosis (3.6% vs. 13.7% in our cohort; χ^2^(1)=17.45, p-value = 2.96⋅10^-5^) and MC (median 0.5 [IQR: 0-1], vs. median 1 [IQR: 0-1]; W=68033, p-value = 0.0076). However, they were comparable in Ki-67% (median 3 [IQR: 2-2.5] vs. median 3 [1-5]; W=59029, p-value=0.447). In addition, the two samples were comparable in terms of patients’ age and sex (see Table 1 in Mathian et al. [5]). Clustering was applied directly on the independent cohort with k=3 (**Supplementary** **Fig. 4**, top). The three clusters had dimensions of 164 (68.8%), 62 (24.5%), and 27 (10.7%), respectively for Clusters 1 to 3. The obtained clusters were semantically comparable to those obtained in our cohort, in the sense that Cluster 1 had the lowest values of Ki-67%, MC, and necrosis presence (and it was also the most populous), while Cluster 3 had the worst conditions, and Cluster 2 was in an intermediate position (**Supplementary** **Table 6**). There were 12 deaths, of which 2, 4, and 6 occurred within Clusters 1 to 3, respectively. The KM curves for the obtained clusters (**Supplementary** **Fig. 5**) had different survival rates (overall log-rank test: χ^2^(2)=35.2, p-value=2⋅10^-8^), particularly in the pairwise comparisons between Clusters (Cl. 1 vs. Cl. 2: p-value=0.0072; Cl. 1 vs. Cl. 3 p-value=2⋅10^-8^; Cl. 2 vs. Cl. 3 p-value=0.0097; all p-values were adjusted by Holm’s method). In the subgroup with available RFS information, 17 relapses occurred, of which 2, 8, and 7 were in Clusters 1 to 3, respectively; 4 deaths occurred before relapse. Crude incidence rates (**Supplementary Fig. 6**) differed across the three clusters (p-value = 2.56⋅10^-6^). Pairwise comparisons suggested that Cl. 1 relapse rate differed from both Cl. 2 (p-value=1.12⋅10^-4^) and Cl. 3 (p-value=3.05⋅10^-7^), while Cl. 2 and Cl.3 curves were comparable (p-value=0.229).

**CAPTIONS OF SUPPLEMENTARY FIGURES**

**Supplementary Fig. 1.** Scatterplot of Ki-67 versus mitotic count, stratified by necrosis status. Points are jittered, *i.e.*, each point is shifted by a small random amount along both axes, to improve readability (densely overlapping observations become visible). Numbers in parentheses indicate the absolute and relative number of necrosis positive and negative carcinoids.


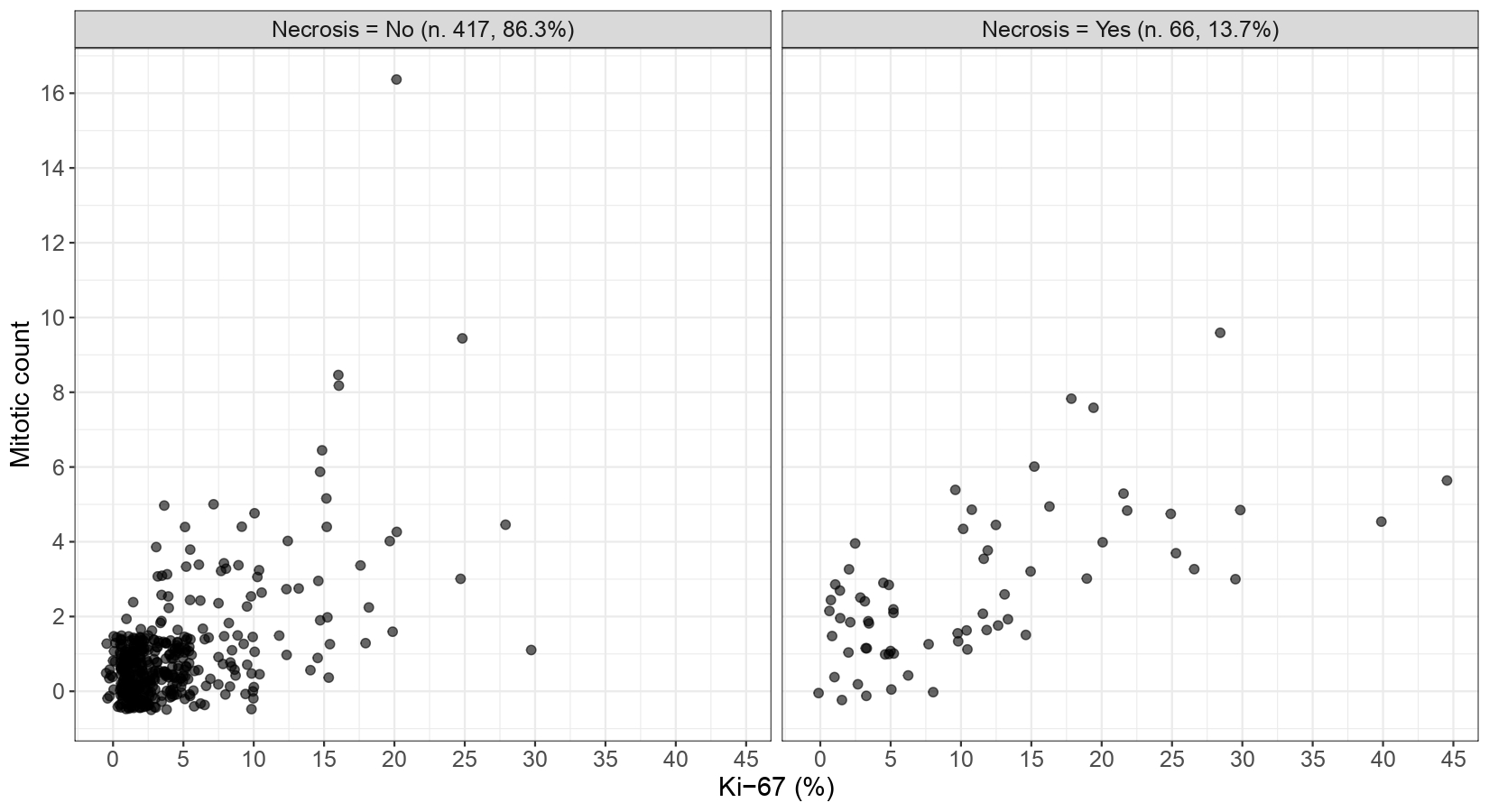


**Supplementary Fig. 2.** Scatterplots of Ki-67 versus mitotic count, separated by necrosis status. Points are colored by the 2-cluster KAMILA’s solution.


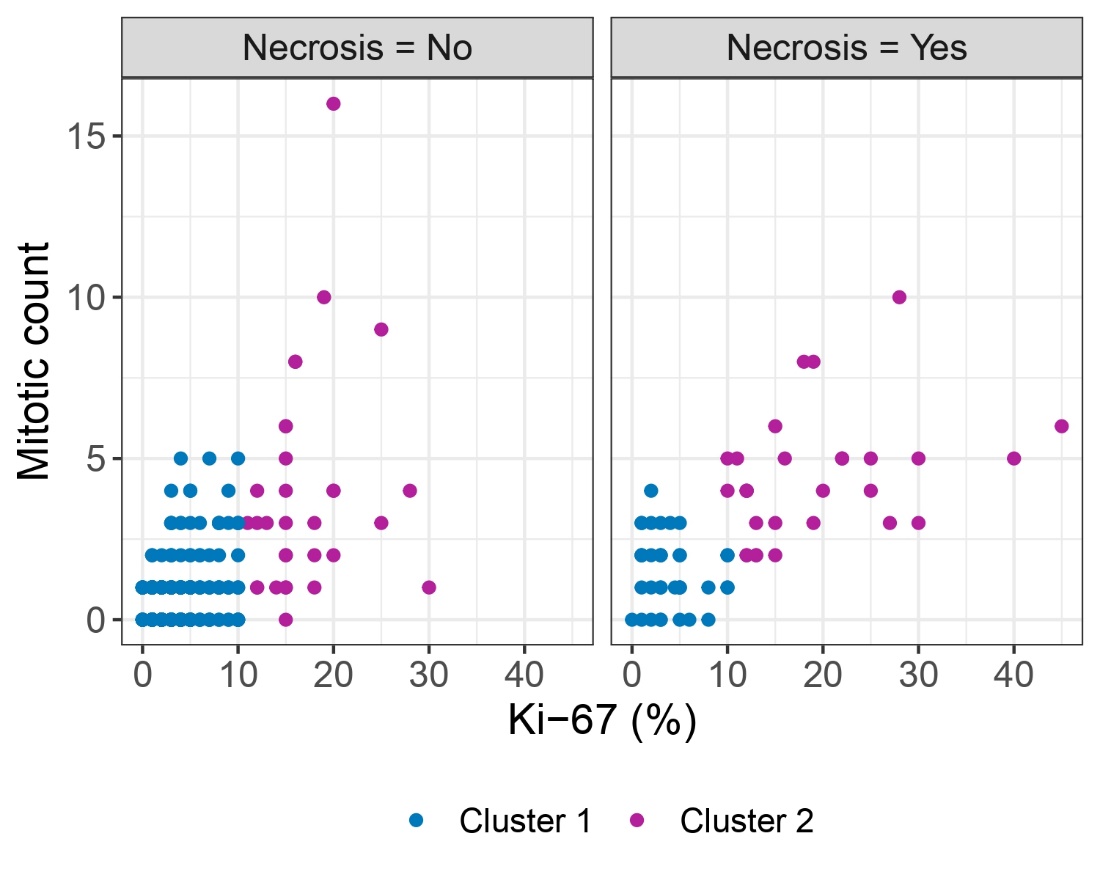


**Supplementary Fig. 3.** Kaplan-Meier survival curves by the current WHO lung NET classification in typical carcinoid (TC) and atypical carcinoid (AC). Censored observations are marked with “+” along the curves. The table reports the number at risk, that is how many patients in each group remain under observation at successive time points; at time 0 the groups start with 121 (AC), 343 (TC) individuals.

**
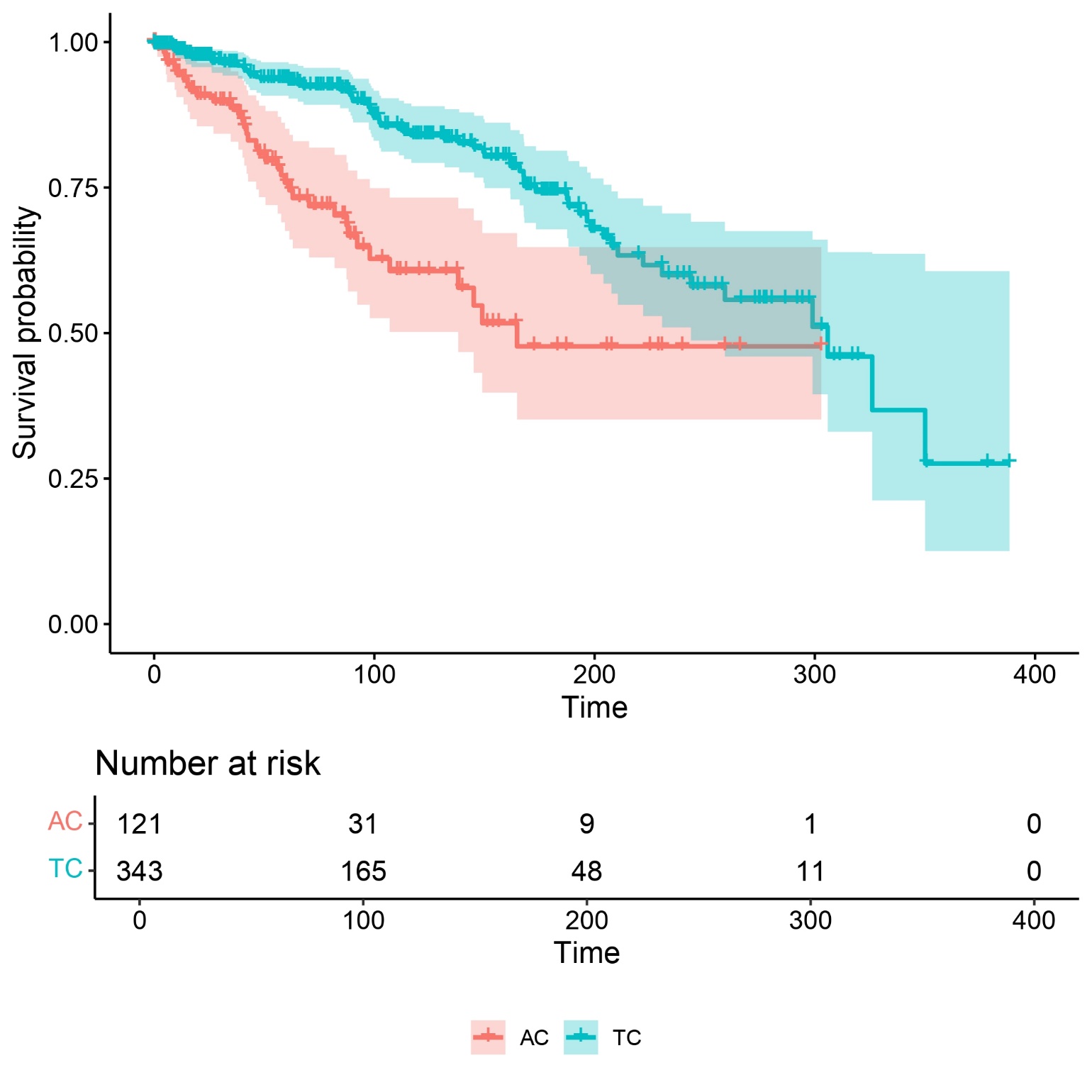
**

**Supplementary Fig. 4.** Scatterplots of data points from the external cohort: Ki-67 (%) is plotted versus mitotic count, separated by necrosis status. Points are colored by the 3-cluster KAMILA’s solution obtained directly on the external dataset (top); by the current WHO classification (middle); and by our proposed classification (bottom).


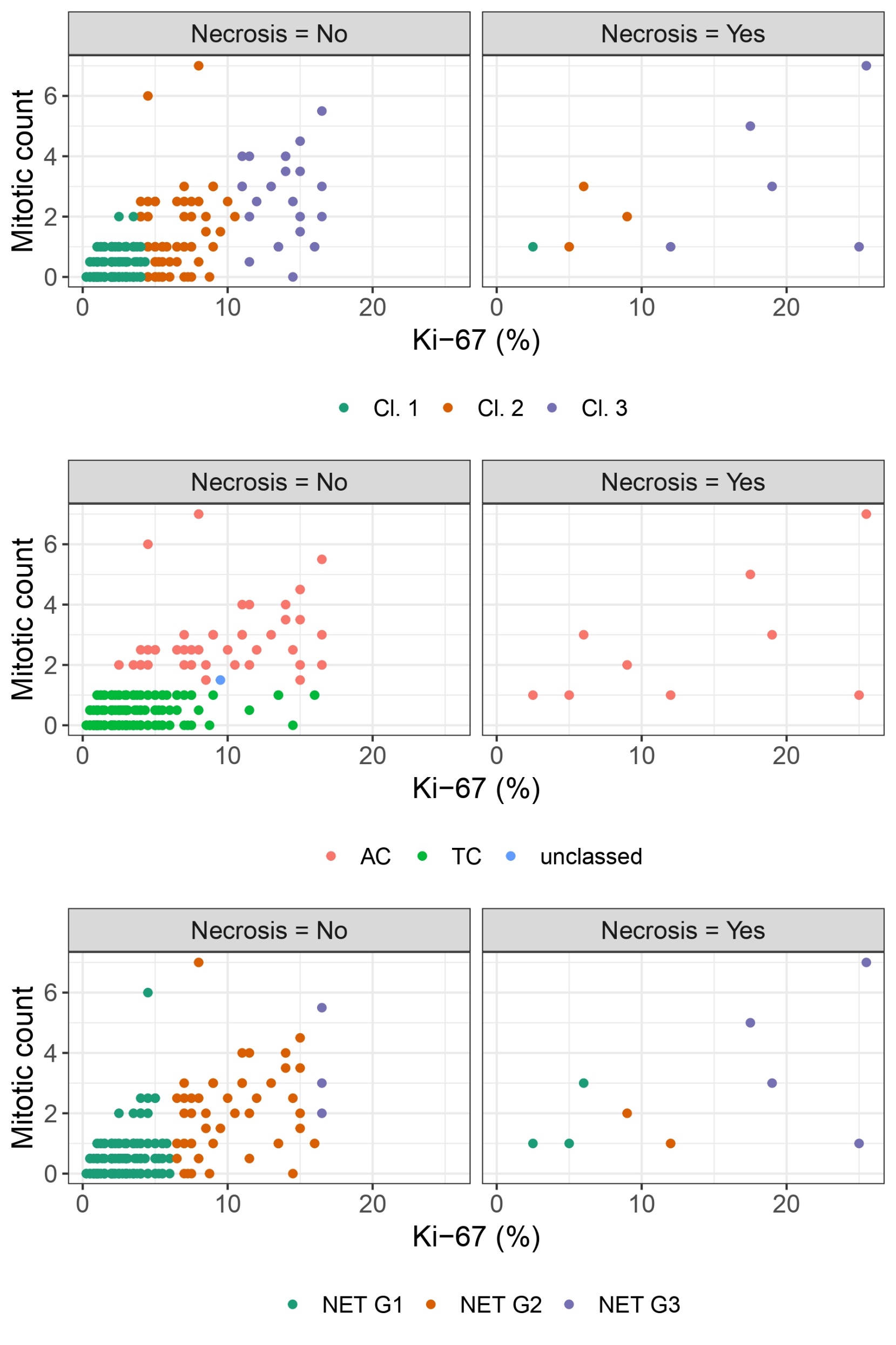


**Supplementary Fig. 5.** Kaplan-Meier survival curves for observations from the external cohort, grouped by the 3-cluster KAMILA’s solution obtained directly on the external dataset. Censored observations are marked with “+” along the curves.


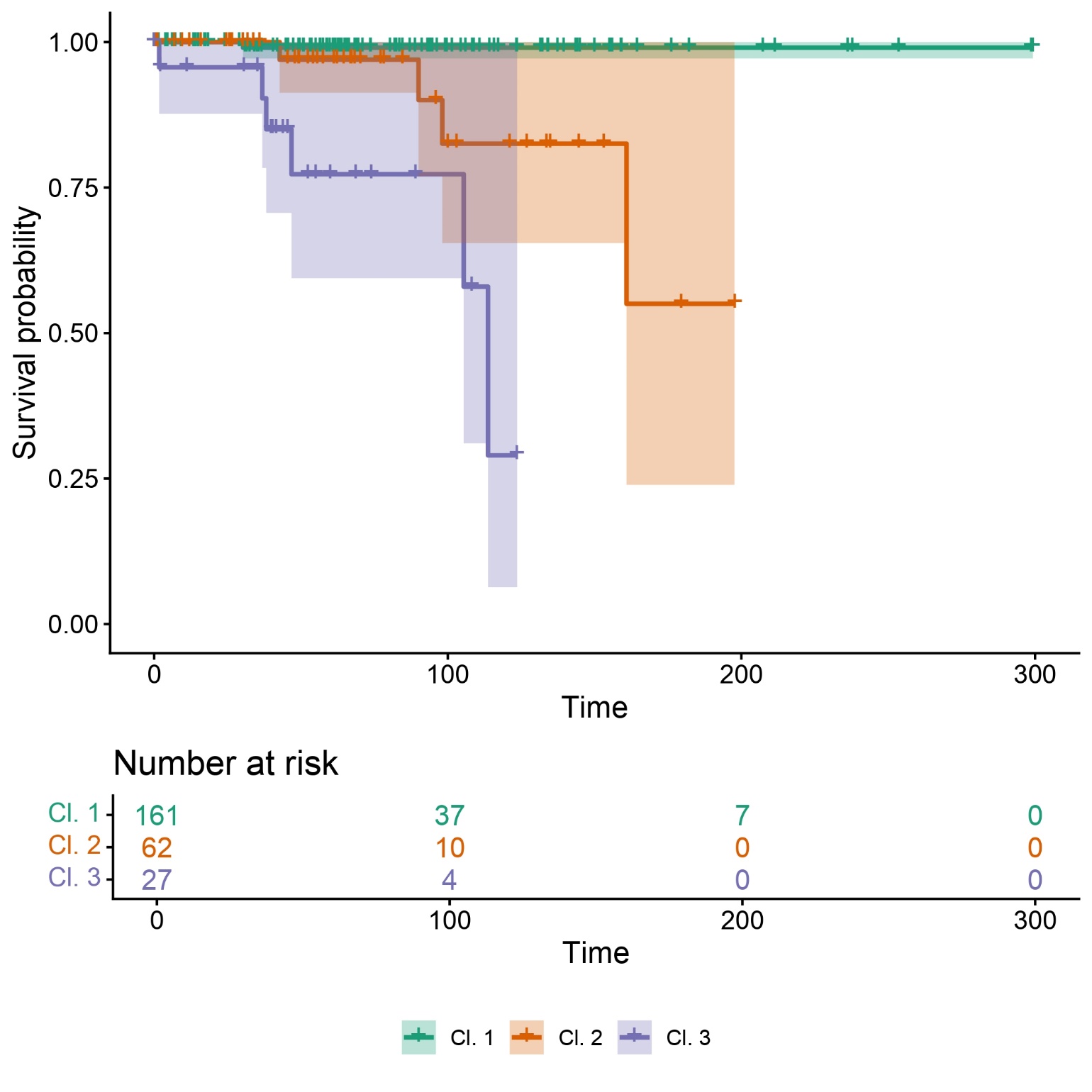


**Supplementary Fig. 6.** Aalen-Johansen crude incidence estimates of relapse within the external cohort, with observations grouped by the 3-cluster KAMILA’s solution obtained directly on the external dataset.

**
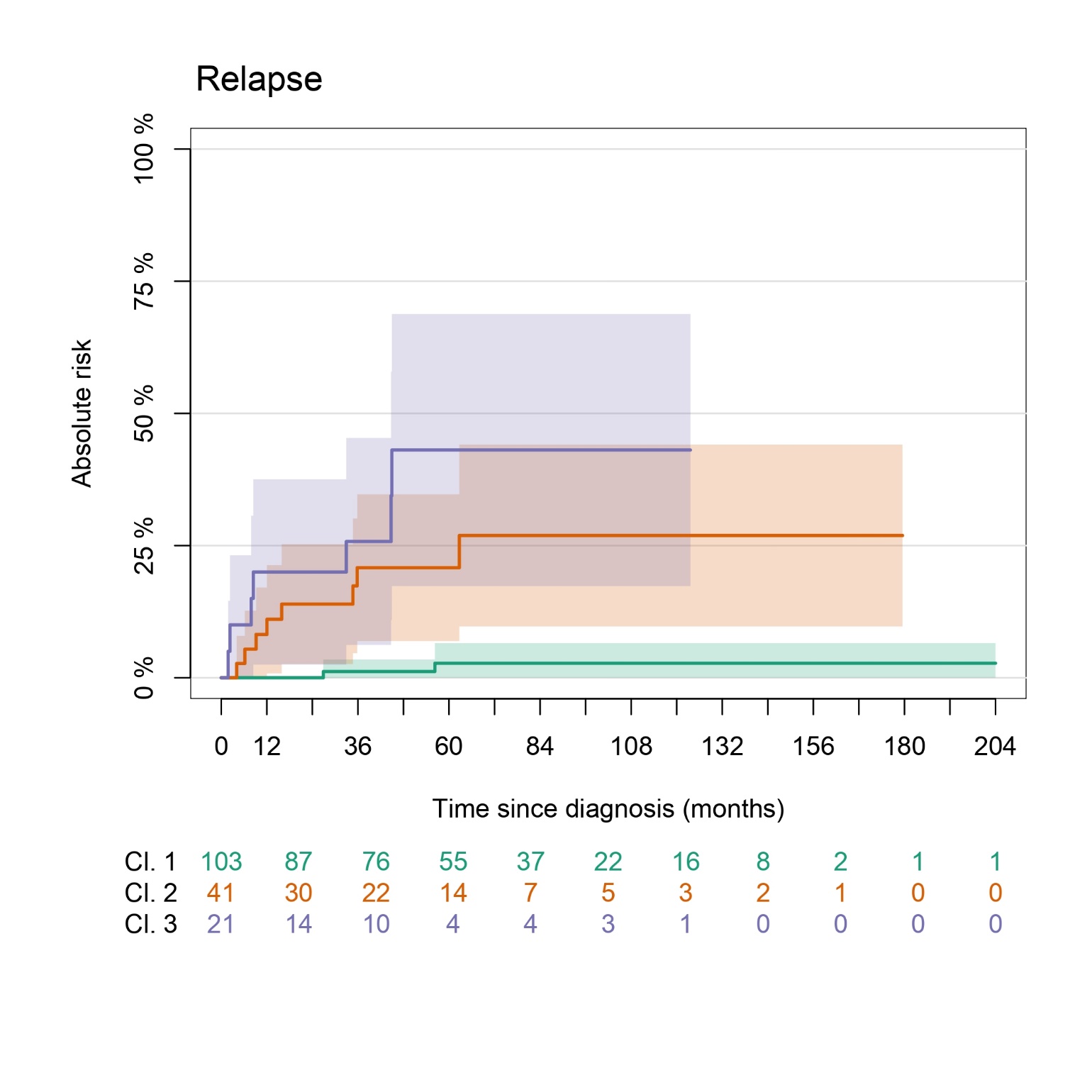
**

**SUPPLEMENTARY TABLES**

**Supplementary Table 1.** Patients’ and tumor characteristics.

|  | **Overall (N=483)** |
| --- | --- |
| **Gender** |  |
| Female, n. (%) | 303 (62.7%) |
| Male, n. (%) | 180 (37.3%) |
| **Age (years)** |  |
| Mean (SD) | 59.31 (14.56) |
| Median [Q1, Q3] | 62.59 [51.71, 69.69] |
| Min - Max | 14.72 - 84.11 |
| **Status** |  |
| NED, n. (%) | 342 (70.8%) |
| AWD, n. (%) | 19 (3.9%) |
| DOD, n. (%) | 43 (8.9%) |
| DOC, n. (%) | 59 (12.2%) |
| Missing, n. (%) | 20 (4.1%) |
| **Relapse** |  |
| No, n. (%) | 393 (81.4%) |
| Yes, n. (%) | 58 (12.0%) |
| Missing, n. (%) | 32 (6.6%) |
| **Tumorlets** |  |
| No, n. (%) | 414 (85.7%) |
| Yes, n. (%) | 69 (14.3%) |
| **Multifocality** |  |
| No, n. (%) | 446 (92.3%) |
| Yes, n. (%) | 37 (7.7%) |
| **Laterality** |  |
| Right, n. (%) | 259 (53.6%) |
| Left, n. (%) | 180 (37.3%) |
| Missing, n. (%) | 44 (9.1%) |
| **Location** |  |
| Central, n. (%) | 217 (44.9%) |
| Unspecified, n. (%) | 124 (25.7%) |
| Peripheral, n. (%) | 142 (29.4%) |
| **Dimension (cm)** |  |
| Mean (SD) | 2.453 (1.456) |
| Median [Q1, Q3] | 2.050 [1.5, 3] |
| Min - Max | 0.5000 - 8.500 |
| Missing, n. (%) | 3 (0.6%) |
| **pT** |  |
| 1a, n. (%) | 44 (9.1%) |
| 1b, n. (%) | 169 (35.0%) |
| 1c, n. (%) | 119 (24.6%) |
| 2a, n. (%) | 65 (13.5%) |
| 2b, n. (%) | 24 (5.0%) |
| 3, n. (%) | 33 (6.8%) |
| 4, n. (%) | 29 (6.0%) |
| **pN (8^th^ edition)** |  |
| 0, n. (%) | 380 (78.7%) |
| 1, n. (%) | 66 (13.7%) |
| 2, n. (%) | 27 (5.6%) |
| Missing, n. (%) | 10 (2.1%) |
| **pM (8^th^ edition)** |  |
| 0, n. (%) | 460 (95.2%) |
| M1a, n. (%) | 4 (0.8%) |
| Missing, n. (%) | 19 (3.9%) |
| **pN (9^th^ edition)** |  |
| 0, no. (%) | 380 (78.7%) |
| 1, no. (%) | 66 (13.7%) |
| 2a, no. (%) | 13 (2.7%) |
| 2b, no. (%) | 9 (1.9%) |
| Missing, no. (%) | 15 (3.1%) |
| **Stage (8^th^ edition)** |  |
| IA1, no. (%) | 35 (7.2%) |
| IA2, no. (%) | 142 (29.4%) |
| IA3, no. (%) | 99 (20.5%) |
| IB, no. (%) | 44 (9.1%) |
| IIA, no. (%) | 16 (3.3%) |
| IIB, no. (%) | 81 (16.8%) |
| IIIA, no. (%) | 44 (9.1%) |
| IIIB, no. (%) | 9 (1.9%) |
| IVA, no. (%) | 4 (0.8%) |
| Missing, no. (%) | 9 (1.9%) |
| **Stage (9^th^ edition)** |  |
| IA, no. (%) | 276 (57.1%) |
| IB, no. (%) | 44 (9.1%) |
| IIA, no. (%) | 53 (11.0%) |
| IIB, no. (%) | 50 (10.4%) |
| IIIA, no. (%) | 35 (7.2%) |
| IIIB, no. (%) | 7 (1.4%) |
| IVA, no. (%) | 4 (0.8%) |
| Missing, no. (%) | 14 (2.9%) |
| **STAS** |  |
| No, no. (%) | 317 (65.6%) |
| Yes, no. (%) | 148 (30.6%) |
| Missing, no. (%) | 18 (3.7%) |
| **Vascular invasion** |  |
| Yes, no. (%) | 105 (21.7%) |
| No, no. (%) | 356 (73.7%) |
| Missing, no. (%) | 22 (4.6%) |

*Abbreviations*: no.: number; NED: no evidence of disease; DOD: died of disease; DOC: died of other causes; AWD: alive with disease; STAS: spread through air spaces.

**Supplementary Table 2.** Ki-67 index, mitotic count, and necrosis summary statistics both in the complete sample (overall) and stratified by necrosis presence/absence.

|  | **Overall (N=483)** | **No necrosis (N=417)** | **Necrosis (N=66)** |
| --- | --- | --- | --- |
| **Ki-67 (%)** |  |  |  |
| Mean (SD) | 4.984 (5.993) | 4.075 (4.499) | 10.730 (9.990) |
| Median [Q1, Q3] | 3.000 [1, 5] | 2.000 [1, 5] | 9.000 [3, 15] |
| Min - Max | 0 - 45.00 | 0 - 30.00 | 0 - 45.00 |
| Not Assessable, n. (%) | 1 (0.2%) | 1 (0.2%) | 0 (0%) |
| **Mitotic count (per 2 mm^2^)** |  |  |  |
| Mean (SD) | 1.180 (1.716) | 0.931 (1.507) | 2.758 (2.091) |
| Median [Q1, Q3] | 1.000 [0, 1] | 1.000 [0, 1] | 2.000 [1, 4] |
| Min - Max | 0 - 16.00 | 0 - 16.00 | 0 - 10.00 |
| **Necrosis** |  |  |  |
| No, no. (%) | 417 (86.3%) |  |  |
| Yes, no. (%) | 66 (13.7%) |  |  |

**Supplementary Table 3.** Cluster’ centers using the KAMILA algorithm, seeking for 2 clusters (“Cl.”), for the variables of interest and comparison with the corresponding WHO classification.

|  | **Cl. 1 (N=423)** | **Cl. 2 (N=60)** |
| --- | --- | --- |
| **Ki-67 (%)** |  |  |
| Mean (SD) | 3.15 (2.51) | 18.17 (7.10) |
| Median [Q1, Q3] | 2 [1, 5] | 15 [13, 20] |
| Min - Max | 0 - 10.00 | 10 – 45 |
| **Mitotic count (per 2 mm^2^)s** |  |  |
| Mean (SD) | 0.76 (0.93) | 4.13 (2.78) |
| Median [Q1, Q3] | 1 [0, 1] | 4 [2, 5] |
| Min - Max | 0 - 10 | 0 – 16 |
| **Necrosis** |  |  |
| No, n. (%) | 386 (91.3%) | 31 (51.7%) |
| Yes, n. (%) | 37 (8.7%) | 29 (48.3%) |
| **WHO Lung NET classification** |  |  |
| AC, n. (%) | 73 (17.3%) | 52 (86.7%) |
| TC, n. (%) | 350 (82.7%) | 8 (13.3%) |

AC: atypical carcinoid; TC: typical carcinoid.

**Supplementary Table 4.** Probabilities for overall survival across the three clusters at 12, 36, and 60 months from diagnosis.

__________________________________________________________________________________

|  | **Overall survival probability** | | |
| --- | --- | --- | --- |
|  | **12-months (95% CI)** | **36-months (95% CI)** | **60-months (95% CI)** |
| **NET G3** | 94.7% (85.2-100%) | 78.6% (62.0-100%) | 33.9% (16.6-69.2%) |
| **NET G2** | 97.1% (93.3-100%) | 93.6% (87.7-100%) | 87.8% (79.6-96.8%) |
| **NET G1** | 97.7% (96.1-99.3%) | 95.4% (93.2-97.7%) | 92.5% (89.6-95.5%) |

___________________________________________________________________________

**Supplementary Table 5.** Results from multivariable Cox’s regression model with cluster labels’, sex, and age assessing overall survival.

_____________________________________________

|  | **HR** | **95% CI** | **p-value** |
| --- | --- | --- | --- |
| Cluster’ labels |  |  |  |
| NET G3 (vs. NET G2) | 1.55 | 0.73, 3.29 | 0.255 |
| NET G1 (vs. NET G2) | 0.57 | 0.34, 0.96 | 0.036 |
| Gender |  |  |  |
| Male (vs. Female) | 2.02 | 1.34, 3.05 | 0.001 |
| Age | 1.09 | 1.06, 1.11 | 1.68⋅10^-13^ |
| HR = Hazard Ratio, CI = Confidence Interval | | | |

**Supplementary Table 6.** Cluster’ centers using the KAMILA algorithm directly on the external cohort observations, seeking for 3 clusters (“Cl.”), for the variables of interest and comparison with the corresponding WHO lung NET classification.

|  | **Cl. 1 (N=164)** | **Cl. 2 (N=62)** | **Cl. 3 (N=27)** | **Overall (N=253)** |
| --- | --- | --- | --- | --- |
| **Ki-67%** |  |  |  |  |
| Mean (SD) | 2.105 (1.012) | 6.368 (1.663) | 14.85 (3.687) | 4.510 (4.336) |
| Median [Q1, Q3] | 2.000 [1, 3] | 6.000 [5, 7.5] | 14.50 [12, 16.25] | 3.000 [2, 5.5] |
| Min - Max | 0.2500 - 4.300 | 4.000 - 10.50 | 11.00 - 25.50 | 0.2500 - 25.50 |
| **Mitotic count (per 2 mm^2^)** |  |  |  |  |
| Mean (SD) | 0.3232 (0.4317) | 1.419 (1.334) | 2.741 (1.655) | 0.8498 (1.215) |
| Median [Q1, Q3] | 0 [0, 0.5] | 1.000 [0.5, 2] | 3.000 [1.25, 3.75] | 0.5000 [0, 1] |
| Min - Max | 0 - 2.000 | 0 - 7.000 | 0 - 7.000 | 0 - 7.000 |
| **Necrosis** |  |  |  |  |
| No, n. (%) | 163 (99.4%) | 59 (95.2%) | 22 (81.5%) | 244 (96.4%) |
| Yes, n. (%) | 1 (0.6%) | 3 (4.8%) | 5 (18.5%) | 9 (3.6%) |
| **Diagnosis WHO** |  |  |  |  |
| AC, n. (%) | 3 (1.8%) | 25 (40.3%) | 22 (81.5%) | 50 (19.8%) |
| TC, n. (%) | 161 (98.2%) | 36 (58.1%) | 5 (18.5%) | 202 (79.8%) |
| Unclassified, n. (%) | 0 (0%) | 1 (1.6%) | 0 (0%) | 1 (0.4%) |

**REFERENCES**

1. Cormack RM (1971) A Review of Classification. J R Stat Soc Ser A 134:321. https://doi.org/10.2307/2344237

2. Foss A, Markatou M, Ray B, Heching A (2016) A semiparametric method for clustering mixed data. Mach Learn 105:419–458. https://doi.org/10.1007/s10994-016-5575-7

3. Foss AH, Markatou M (2018) kamila: Clustering Mixed-Type Data in R and Hadoop. J Stat Softw 83:1–44. https://doi.org/10.18637/jss.v083.i13

4. Hothorn T, Zeileis A (2015) partykit: A modular toolkit for recursive partitioning in R. 16:3905–3909

5. Mathian É, Drouet Y, Sexton-Oates A, et al (2024) Assessment of the current and emerging criteria for the histopathological classification of lung neuroendocrine tumours in the lungNENomics project. ESMO Open 9:103591. https://doi.org/10.1016/j.esmoop.2024.103591
